# The microtubule signature in cardiac disease: etiology, disease stage, and age dependency

**DOI:** 10.1007/s00360-023-01509-1

**Published:** 2023-08-29

**Authors:** Sıla Algül, Larissa M. Dorsch, Oana Sorop, Aryan Vink, Michelle Michels, Cristobal G. dos Remedios, Michiel Dalinghaus, Daphne Merkus, Dirk J. Duncker, Diederik W. D. Kuster, Jolanda van der Velden

**Affiliations:** 1 Department of Physiology, Amsterdam UMC, Vrije Universiteit Amsterdam, Amsterdam Cardiovascular Sciences, O2 Building, De Boelelaan 1117, 1081HV Amsterdam, The Netherlands; 2 Division of Experimental Cardiology, Department of Cardiology, Thoraxcenter, Erasmus University Medical Center, Rotterdam, The Netherlands; 3 Department of Pathology, University Medical Center, Utrecht, The Netherlands; 4 Mechanobiology Laboratory at Victor Chang Cardiac Research Institute, Darlinghurst, NSW 2010 Australia; 5 Department of Pediatric Cardiology, Sophia Children’s Hospital, Erasmus University Medical Center, Rotterdam, The Netherlands

**Keywords:** Microtubules, Desmin, Cardiomyopathy, Hypertrophic, Cardiomyopathy, Dilated, Myocytes, Cardiac, Cardiac remodeling, Ventricular

## Abstract

Employing animal models to study heart failure (HF) has become indispensable to discover and test novel therapies, but their translatability remains challenging. Although cytoskeletal alterations are linked to HF, the tubulin signature of common experimental models has been incompletely defined. Here, we assessed the tubulin signature in a large set of human cardiac samples and myocardium of animal models with cardiac remodeling caused by pressure overload, myocardial infarction or a gene defect. We studied levels of total, acetylated, and detyrosinated α-tubulin and desmin in cardiac tissue from hypertrophic (HCM) and dilated cardiomyopathy (DCM) patients with an idiopathic (*n* = 7), ischemic (*n* = 7) or genetic origin (*n* = 59), and in a pressure-overload concentric hypertrophic pig model (*n* = 32), pigs with a myocardial infarction (*n* = 28), mature pigs (*n* = 6), and mice (*n* = 15) carrying the HCM-associated *MYBPC3*_2373insG_ mutation. In the human samples, detyrosinated α-tubulin was increased 4-fold in end-stage HCM and 14-fold in pediatric DCM patients. Acetylated α-tubulin was increased twofold in ischemic patients. Across different animal models, the tubulin signature remained mostly unaltered. Only mature pigs were characterized by a 0.5-fold decrease in levels of total, acetylated, and detyrosinated α-tubulin. Moreover, we showed increased desmin levels in biopsies from NYHA class II HCM patients (2.5-fold) and the pressure-overload pig model (0.2–0.3-fold). Together, our data suggest that desmin levels increase early on in concentric hypertrophy and that animal models only partially recapitulate the proliferated and modified tubulin signature observed clinically. Our data warrant careful consideration when studying maladaptive responses to changes in the tubulin content in animal models.

**Graphical Abstract:**

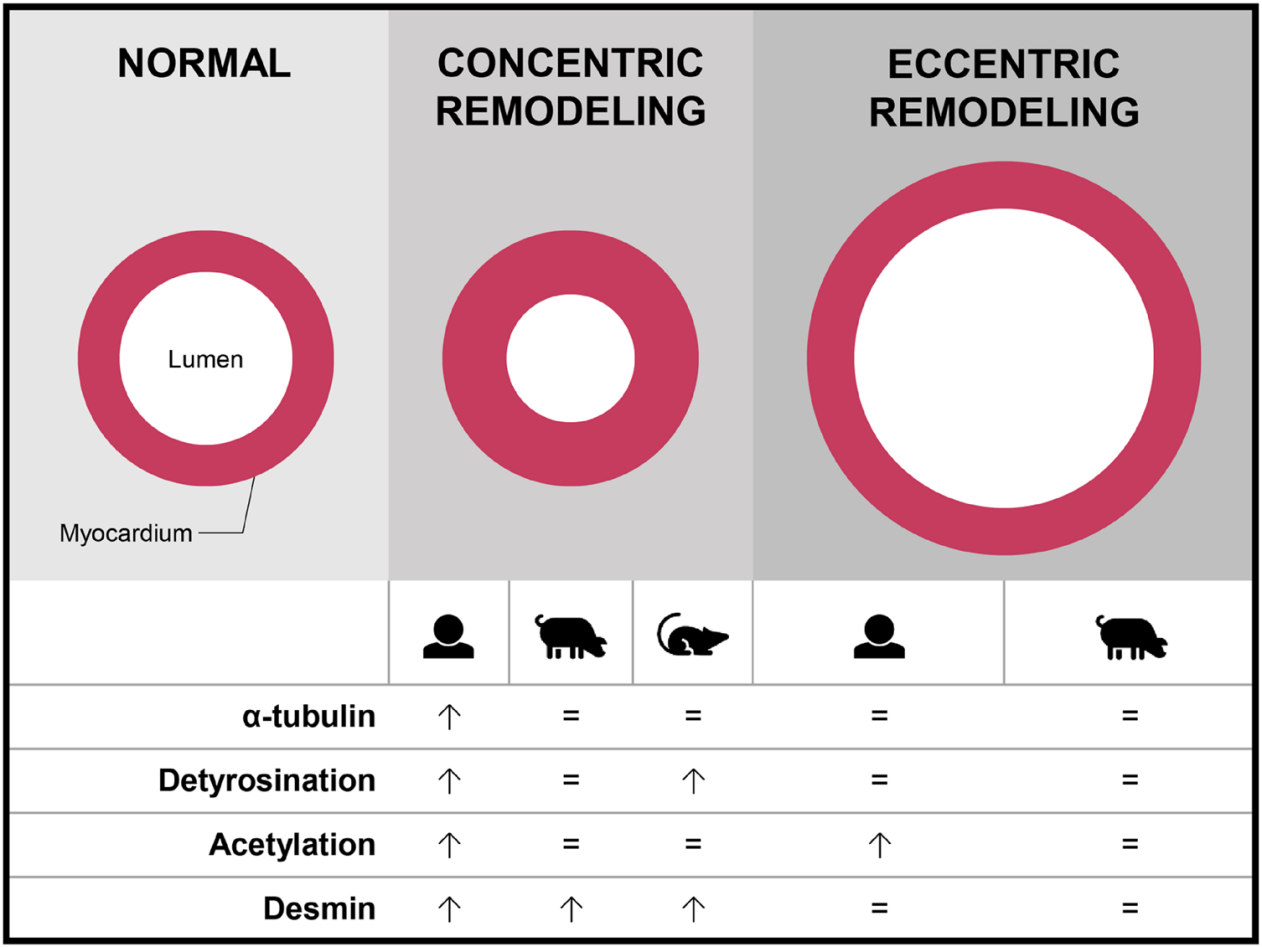

**Supplementary Information:**

The online version contains supplementary material available at 10.1007/s00360-023-01509-1.

## Introduction

Cardiac disease has diverse etiologies ranging from valvular defects and hypertension to an ischemic insult or genetic defect. Aortic stenosis and hypertension both cause concentric remodeling of the heart, which is characterized by an increase of the left ventricular (LV) wall thickness to a greater extent than the volume of the LV cavity (Gjesdal et al. 2011). On the other hand, an ischemic insult (myocardial infarction) causes eccentric remodeling, in which the heart dilates and the wall becomes thin (Gaasch and Zile 2011; Gjesdal et al. 2011). Gene defects can cause either concentric or eccentric cardiac remodeling, which are known as hypertrophic (HCM) and dilated cardiomyopathy (DCM), respectively (Gjesdal et al. 2011). Such structural alterations arise from the cytoskeleton, which consists of sarcomeric and non-sarcomeric structures (Sequeira et al. 2014). The latter category, to which microtubules and intermediate filaments belong, have a pronounced effect on the morphological integrity of cardiomyocytes, yet little is known about their position in the onset and progression of cardiac disease (Sequeira et al. 2014).

The view that cytoskeletal alterations may play a pivotal role in cardiac remodeling is increasingly becoming an important aspect of understanding cardiac function. The Prosser group has previously reported proliferation of microtubules and intermediate filaments, and of the post-translational modification detyrosination of microtubules in end-stage failing human hearts (Caporizzo et al. 2018; Chen et al. 2018; Robison et al. 2016). Desmin, an intermediate filament that bundles along the Z-disc (Konieczny et al. 2008), was recently identified as a sarcomeric microtubule anchor (Konieczny et al. 2008; Robison et al. 2016). By cross-linking with microtubules in a detyrosination-dependent manner (Gurland and Gundersen 1995; Liao and Gundersen 1998), desmin was reported to be increased in heart failure (HF) patients and using septal myectomy samples of HCM patients (Chen et al. 2018; Schuldt et al. 2021). Continuing this work, we have observed increases in the acetylation (ac) of α-tubulin, another post-translational modification of microtubules, in HCM samples with *MYBPC3* mutations (Dorsch et al. 2019; Gilbert et al. 1996; Harris et al. 2002).

Based on these observations, the aim of this paper was twofold—first, to study whether cytoskeletal remodeling is dependent on cardiac remodeling. Second, to assess whether common animal models of HF recapitulate the cytoskeletal remodeling observed in patients. We thereto studied the microtubule network in cardiac tissue from HCM and DCM patients with different disease stages and whose cardiomyopathies had an idiopathic, ischemic or genetic origin. To define changes in the tubulin signature and its post-translational modifications at an early stage, we studied the microtubule network in different animal models. More specifically, we compared 4-month-old sham-operated pigs to aortic banding (AoB)-treated pigs of the same age, a model that has been used to study pressure-overload concentric hypertrophy, and to pigs with a myocardial infarction (MI) to examine eccentric remodeling (Marshall et al. 2013; van Boven et al. 2018; Xiong et al. 2015). In addition, sham-operated, 4-month-old pigs were compared to 18-month-old pigs to evaluate the effect of aging (van Essen et al. 2018). To address the effect of a sarcomeric mutation, we used septal tissue from a mouse model carrying a Dutch founder mutation in the gene encoding myosin binding protein C (Schuldt et al. 2021). Collectively, our data support the notion that the cytoskeleton is altered independent of cardiac remodeling and maintains a rather disease-specific progression.

## Materials and methods

### Human cardiac samples

Cardiac samples were studied from adult patients with obstructive HCM (*n* = 38; 26 males, 12 females; mean age 48 ± 16 years), end-stage HCM (*n* = 7; 3 males, 4 females; mean age 44 ± 13 years), ischemic heart disease (ISHD; *n* = 7; 6 males, 1 female; mean age 56 ± 6 years), idiopathic DCM (IDCM; *n* = 7; 4 males, 1 females; mean age 51 ± 4 years), end-stage DCM with an identified DCM-causing mutation (DCM_end_; *n* = 7; 5 males, 2 females; mean age 56 ± 8 years), and 7 pediactric patients with DCM (DCM_ped_; 2 males, 3 females; mean age 10 ± 3 years). Samples from eight healthy non-failing donors (5 males, 3 females; mean age 47 ± 11 years) served as controls. Parameters of all HCM and DCM individuals and donors are summarized in Table 1. All samples were obtained after written informed consent from each patient prior to surgery and from the patients’ or donors’ next of kin. Some of these samples were analyzed in previous studies (Dorsch et al. 2019; Schuldt et al. 2021).

**Table 1:** Characteristics of HCM, DCM, and non-failing individuals

Number	Group	Remark	Affected genes	Sex (F/M)	Age	FS(%)	LVEF	LVEDD	LVESD
8	Ctrl	–	–	3 F, 5 M	47 ± 11	–	–	–	–
38	HCM_St II_	–	19 *MYBPC3*, 10 *MYH7*, 2 *MYL2*, 4 *TNNI3*, 3 *TNNT2*	12 F, 26 M	48 ± 16	48 ± 11 (*n* = 15)	81 ± 15 (*n* = 2)	43 ± 5 (*n* = 31)	22 ± 5 (*n* = 15)
7	HCM_St IV_	–	1 *MYBPC3*, 5 *MYH7*, 1 *TNNT2*	4 F, 3 M	44 ± 13	–	–	57 ± 9 (*n* = 6)	–
7	ISHD	–	–	1 F, 6 M	56 ± 6 (*n* = 6)	–	24 ± 6 (*n* = 6)	72 (*n* = 1)	66 (*n* = 1)
7	IDCM	–	–	1 F, 4 M (*n* = 5)	51 ± 4 (*n* = 5)	–	18 ± 6 (*n* = 3)	79 ± 6 (*n* = 2)	67 ± 11 (*n* = 2)
7	DCM_end_	5 DCM, 2 DCM/ACM	1 *DSP*, 1 *LMNA* & *TTN*, 2 *PLN*, 1 *TNNT2*, 2 *TTN*	2 F, 5 M	56 ± 8	–	–	–	–
7	DCM_ped_	6 DCM, 1 RCM	1 *DES*, 1 *MYH7*, 1 *NKX2.5*, 1 *TPM1*	3 F, 2 M (*n* = 5)	10 ± 3 (*n* = 5)	13 ± 7 (*n* = 6)	26 ± 14 (*n* = 6)	6 ± 4 (*n* = 6)	9 ± 3 (*n* = 6)

*F* female, *M* male, *Age* age at septal myectomy/heart transplantation, *FS* fractional shortening, *LVEF* LV ejection fraction, *LVEDD* LVend-diastolic dimension, *LVESD* LV end-systolic dimension, *Ctrl* healthy donor hearts, *HCM*_*St II*_ early HCM, *HCM*_*St IV*_ end-stage HCM, *ISHD* ischemic heart disease, *IDCM* idiopathic DCM, *DCM*_*end*_ adult end-stage genetic DCM, *DCM*_*ped*_ pediatric DCM, *ACM* arrhythmogenic cardiomyopathy, ^#^ LVEDD and LVESD expressed as Z score for body surface are in pediatric samples

Interventricular septum tissue of HCM patients was collected during myectomy surgery to relieve LV outflow tract obstruction. Cardiac tissue from end-stage HCM patients was obtained during heart transplantation surgery. All HCM samples were approved by the local ethics board of the Erasmus Medical Center Rotterdam, the Netherlands (protocol number MEC-2010-40). LV tissue from ISHD and IDCM samples were acquired during transplantation from the University of Sydney, Australia, with the ethical approval of the Human Research Ethics Committee #2012/2814. Familial DCM samples were acquired during transplantation from the Biobank of the University Medical Center Utrecht, the Netherlands and approved by the Biobank Research Ethics Committee, Utrecht, the Netherlands (protocol number WARB 12/387). Pediactric DCM samples were approved by the local ethics board of the Erasmus Medical Center Rotterdam, the Netherlands (protocol number MEC-2015-233). Five of these samples were obtained during transplantation and two samples were post-mortem. Donor samples were obtained from the Sydney Heart Bank, Australia with the ethical approval of the Human Research Ethics Committee (HREC Univ Sydney 2012/030).

### Mice: MYBPC3-targeted mouse model

Lysates derived from LV myocardial/septal tissue from mice carrying the Dutch founder mutation in the *MYBPC3* gene (c.2373 InsG) in one allele or both alleles were utilized for the western blotting experiments. Tissue was harvested previously (Schuldt et al. 2021). Animal care and sacrifice were conducted in accordance with the Guide for the Animal Care and Use Committee of the VU University Medical Center (VUmc) and with approval of the Animal Care Committee of the VUmc Amsterdam, the Netherlands. A total of *n* = 15 mice of either sex, 20–28 weeks of age were included in this study. Heterozygous (HET; *n* = 5) mice carrying the point mutation in one allele and homozygous (HOM; *n* = 5) *MYBPC3*-targeted mice were compared to WT littermates. Parameters of all mice are summarized in Supplemental Table 1.

### Pig models

We were able to use a large set of biobanked LV tissue samples that were collected in previous studies (Duncker et al. 2009; Kats et al. 2000; Kuster et al. 2011; van Boven et al. 2018; van Essen et al. 2018). All studies in pigs were performed in accordance with the Council of Europe Convention (ETS123) and the Directive (2010/63/EU) for the protection of vertebrate animals used for experimental and other scientific purposes, and with approval of the Animal Care Committee of Erasmus University Medical Center Rotterdam, the Netherlands. Experiments were performed in Yorkshire × Landrace pigs. Concentric remodeling was induced in pigs (eight males, eight females) by aortic banding (AoB) and compared to sham-operated pigs (eight males, eight females). Sham-operated and AoB-treated pigs were sacrificed at either 3 weeks (*n* = 16, 8 for both study conditions) or 8 weeks (*n* = 16, 8 for both study conditions) after surgery. Eccentric remodeling was induced in a similar setup. Pigs either underwent a sham operation (five males, nine females) or a myocardial infarction (MI, seven males, seven females) and were sacrificed at either 3 weeks (*n* = 16, 8 per study condition) or 6 weeks after surgery (*n* = 12, 6 per study condition). Cardiac samples (*n* = 6) from 18-month-old sows with a high body weight (BW) (170–216 kg; (van Essen et al. 2018)) were used for an age-dependent comparison to the sham-operated, 4-month-old pigs originally assigned to the AoB study. Parameters of all pigs are summarized in Supplemental Table 2.

### Pigs: AoB treatment, induction of MI, and sham procedures

In pigs allocated for the concentric remodeling model, a left thoracotomy was performed and the proximal ascending aorta was dissected free. In AoB pigs, a band was then placed around the aorta, resulting in a systolic pressure gradient of 68 ± 3 mmHg (van Boven et al. 2018). Subsequently, the chest was closed and the animals were allowed to recover.

The surgical procedure for pigs assigned to the MI study has been described previously (Duncker et al. 2009; Kuster et al. 2011). Briefly, a left thoracotomy was used to access the left circumflex coronary artery (LCx), which was then dissected and a suture was placed losely around the artery. This suture was removed in sham-operated pigs, while in pigs assigned to the MI group, the suture was closed and the LCx remained permanently ligated.

Prior to sacrifice, 2D echocardiographic recordings were obtained for all animals. LV end-diastolic cross-sectional area (EDA) and end-systolic cross-sectional area (ESA) were determined, and left ventricular ejection fraction (LVEF) was calculated as (EDA − ESA)/EDA×100%. At 3, 6, or 8 weeks after surgery, pigs from all study conditions were re-anesthetized and sacrificed, after which the heart was rapidly excised and weighed. LV biopsies were obtained from the subendocardium of the anterior free wall, snap frozen and stored in liquid nitrogen until further analysis. After removal of the large vessels, total heart weight was determined. Then the atria and right ventricle were dissected and LV weight (LVW) was determined.

### Tissue homogenization

Pulverized frozen tissue was homogenized in 40 μL/mg tissue 1 × reducing sample buffer (106 mmol/L Tris–HCl, 141 mmol/L Tris-base, 2% lithium dodecyl sulfate, 10% glycerol, 0.51 mmol/L ethylenediaminetetraacetic acid, 0.22 mmol/L SERVA Blue G250, 0.18 mmol/L Phenol Red, and 100 mmol/L dithiothreitol) using a glass tissue grinder. Proteins were denatured by heating to 99 °C for 5 min, after which debris was removed by centrifugation at maximum speed for 10 min in a microcentrifuge (Sigma, 1-15 K).

### Electrophoresis and western blots

Equal amounts of protein (10 µg for pig and human samples; 5 µg for mouse samples) were separated on pre-cast SDS-PAGE 4–12% criterion gels (Bio-Rad, Hercules, CA, USA) and transferred onto PVDF membranes (GE Healthcare, Chicago, IL, USA). The membranes were blocked in 5% (w/v) skim milk/1 × TBST (20 mmol/L Tris, 150 mmol/L NaCl, 0.1% (v/v) Tween-20) for 1 h at room temperature and incubated overnight at 4 °C with the following primary antibodies in 3% (w/v) BSA/1 × TBST: mouse anti-acetylated α-tubulin (T7451, Sigma-Aldrich, Saint Louis, MO, USA), rabbit anti-desmin (#5332, Cell Signaling Technology, Danvers, MA, USA), rabbit anti-detyrosinated α-tubulin (ab48389, Abcam, Cambridge, UK), mouse anti-GAPDH (10R-G109b, Fitzgerald Industries International, Acton, MA, USA), rabbit anti-GAPDH (#2118, Cell Signaling), mouse anti-cMyBP-C (sc-137180, Santa Cruz, Dallas, TX, USA), and mouse anti-α-tubulin (T9026, Sigma-Aldrich). Membranes were incubated for 1 h at room temperature with horseradish peroxidase-conjugated secondary antibody (DakoCytomation, Santa Clara, CA, USA), raised in goat, in 3% (w/v) BSA/1 × TBST. Western blots were developed with Amersham ECL prime western blotting detection reagent and images were acquired using Amersham Imager 600 and quantified by densitometry by ImageQuant TL software (all GE Healthcare). To correct for loading differences, protein amounts were expressed relative to GAPDH (Figure S10). To correct for comparing samples on different membranes, a control sample was randomly selected and consistently loaded on every membrane for additional normalization. The ratio of a given post-translational modification over total tubulin was calculated by dividing the normalized value of the post-translational modification by the normalized total tubulin value.

### Statistical analyses

Data were analyzed with SPSS Statistics version 26.0 for Windows (IBM Corporation, Armonk, NY, USA) and GraphPad Prism version 8.2.1 (GraphPad Software Inc., San Diego, CA, USA), the latter of which was also used to create all graphs. Results are expressed as means ± standard errors of the mean (SEM). After data collection, the distribution of each data set was determined by Q-Q plots and then double checked by Kolmogorov–Smirnov and Shapiro–Wilk tests. Normally distributed data were analyzed with unpaired *t* test (comparing 2 groups) and ordinary one-way ANOVA (comparing > 2 groups) with Tukey’s multiple comparisons post hoc test (comparing ≤ 3 groups). To analyze non-parametric data, the Mann–Whitney U test (comparing 2 groups) and the Kruskal–Wallis test with Dunn’s multiple comparisons post hoc test (comparing > 2 groups) were used. A two-sided *P* < 0.05 was considered statistically significant.

## Results

### Defining the tubulin signature in HCM samples from patients with different disease stage

**Fig. 1:**
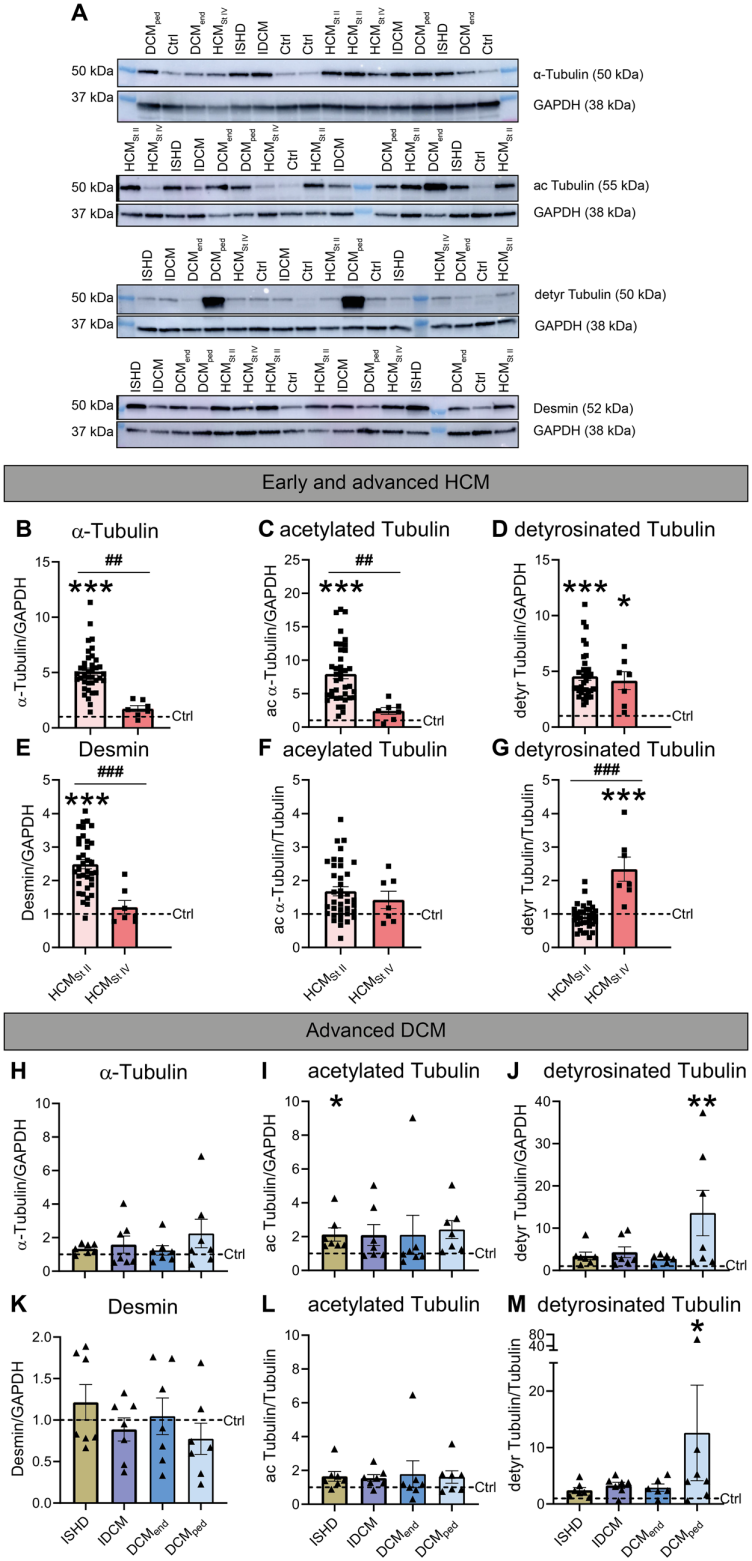
Microtubule remodeling and increased desmin levels are most prominent in early HCM. **A** Representative blot images with uncropped membranes. Lanes that are unlabeled constitute protein ladders. In (**B**–**G**), early (HCM_St II_) and end-stage (HCM_St IV_) HCM samples were compared to controls (Ctrl) and in (**H**–**M**), end-stage DCM samples including ISHD, IDCM, adult (DCM_end_) and pediatric (DCM_ped_) were compared to Ctrl. Quantified levels of (**B**, **H**) α-tubulin, (**C**, **I**) acetylated α-tubulin, (**D**, **J**) detyrosinated α-tubulin, (**E**, **K**) desmin, (**F**, **L**) acetylated α-tubulin normalized to α-tubulin, and (**G**, **M**) detyrosinated α-tubulin normalized to α-tubulin. The average value of the control samples is indicated by the dotted line in the respective scatter plots. HCM samples = filled squares, HCM_St II_ = brighter red bar, HCM_St IV_ = darker red bar, DCM samples = filled triangles, ISHD = brown bar, IDCM = purple bar, DCM_end_ = darker blue bar, DCM_ped_ = brighter blue bar. Each dot in the scatter plots represents an individual sample. * *P* < 0.05, ** *P* < 0.01 and *** *P* < 0.001 versus Ctrl, ^##^ *P* < 0.01 and ^###^ *P* < 0.001 versus HCM_St IV_. Measurements are means ± SEMs

We first sought to define the tubulin signature and desmin levels in cardiac tissue from HCM patients with different disease stages ranging from NYHA class II (HCM_St II_) to end-stage HF (NYHA class IV, HCM_St IV_) and with a genetic origin (Fig. 1). We observed significantly higher levels of α-tubulin and desmin in HCM_St II_ compared to HCM_St IV_ and controls (Fig. 1B and E). In addition, acetylated α-tubulin was significantly higher in HCM_St II_, while detyrosinated α-tubulin was significantly higher in both HCM_St II_ and HCM_St IV_ compared to controls (Fig. 1C, D). Normalization of acetylated and detyrosinated levels to α-tubulin abolished the differences between HCM_StII_ compared to controls, indicating that the increased levels of post-translational modifications coincide with increased microtubules expression at the early HCM disease stage, while the normalized detyrosinated microtubule level was significantly higher in end-stage HCM samples compared to HCM_St II_ and controls (Fig. 1F, G). Categorizing the HCM_St II_ samples into thick (MYH7/MYL2) and thin (TNNI3 and TNNT2) filament samples shows no variation in the microtubular signature (Figure S7).

As a next step, we studied the microtubule network and the respective post-translational modifications of LV samples from patients with eccentric remodeling (Fig. 1H–M). When comparing different end-stage dilated human HF samples, we observed no differences in levels of α-tubulin and desmin compared to controls (Fig. 1H and K). Compared to controls, acetylated α-tubulin was significantly higher in ISHD patients, while detyrosinated α-tubulin and normalized detyrosination levels were higher in pediatric DCM patients (DCM_ped_, Figs. 1I, J, M).

### Tubulin signatures in HET MYBPC3_2373InsG_ mice do not differ from WT mice

**Fig. 2:**
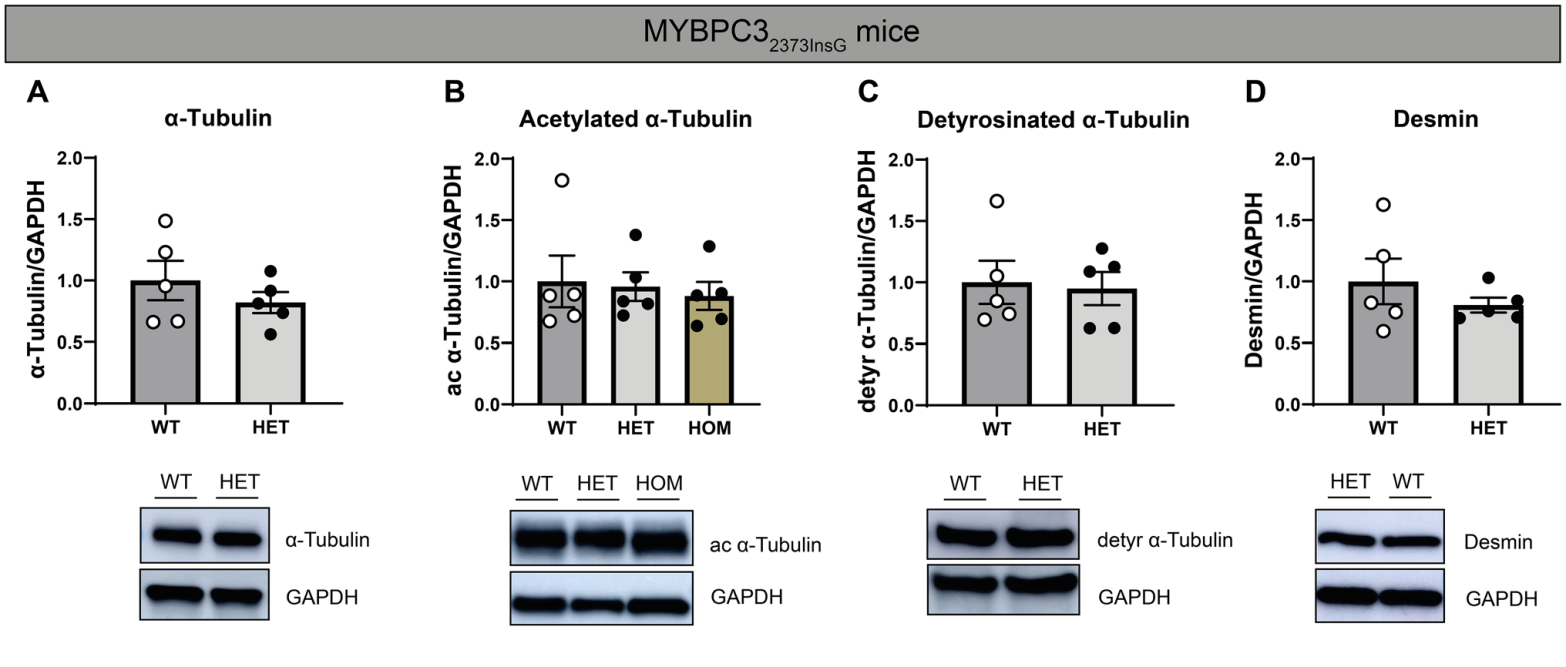
Levels of microtubules and post-translational modifications in HET MYBPC3 mice remain unaltered. **A**–**D** HET mice in addition to HOM mice were compared to WT littermates, with representative western blot images shown accordingly. Lanes that are unlabeled constitute protein ladders. Quantified levels of (**A**) α-tubulin, (**B**) acetylated α-tubulin, (**C**) detyrosinated α-tubulin, (**D**) and desmin. The data displayed for the HOM mice (**A**, **C** and **D**) are derived from a previously published paper of our group and is meant to show that compared to WTs, levels of detyrosinated α-tubulin and desmin are significantly increased, with total levels of α-tubulin remaining unaltered (Schuldt et al. 2021). WT = open circles, HET = filled circles, light gray bar, HOM = filled circles, green bar. Each dot in the scatter plots represents an individual sample. Measurements are mean ± SEM

The c.2373 InsG point mutation in the *MYBPC3* gene is a Dutch founder mutation and accounts for approximately 23% of the mutations found in the Dutch HCM patient population. Much like MYBPC3_2373InsG_ patients at the time of myectomy, HOM MYBPC3_2373InsG_ mice present with concentric hypertrophy and increased cardiac levels of detyrosinated α-tubulin and desmin (Schuldt et al. 2021). To determine if the tubulin signature is altered in HET mice (which reflect the genetic background of most HCM patients, i.e., a heterozygous gene mutation), studies were expanded to compare HET and HOM mice to WT. As is shown in Fig. 2A–D and Figure S8 (normalization to total tubulin levels), compared to WT littermates, we observed no changes in levels of α-tubulin, detyrosinated α-tubulin, and desmin in HET mice. Likewise, levels of acetylated α-tubulin in both HET and HOM mice did not differ from WTs.

### Increased levels of desmin in pigs with concentric cardiac remodeling

**Fig. 3:**
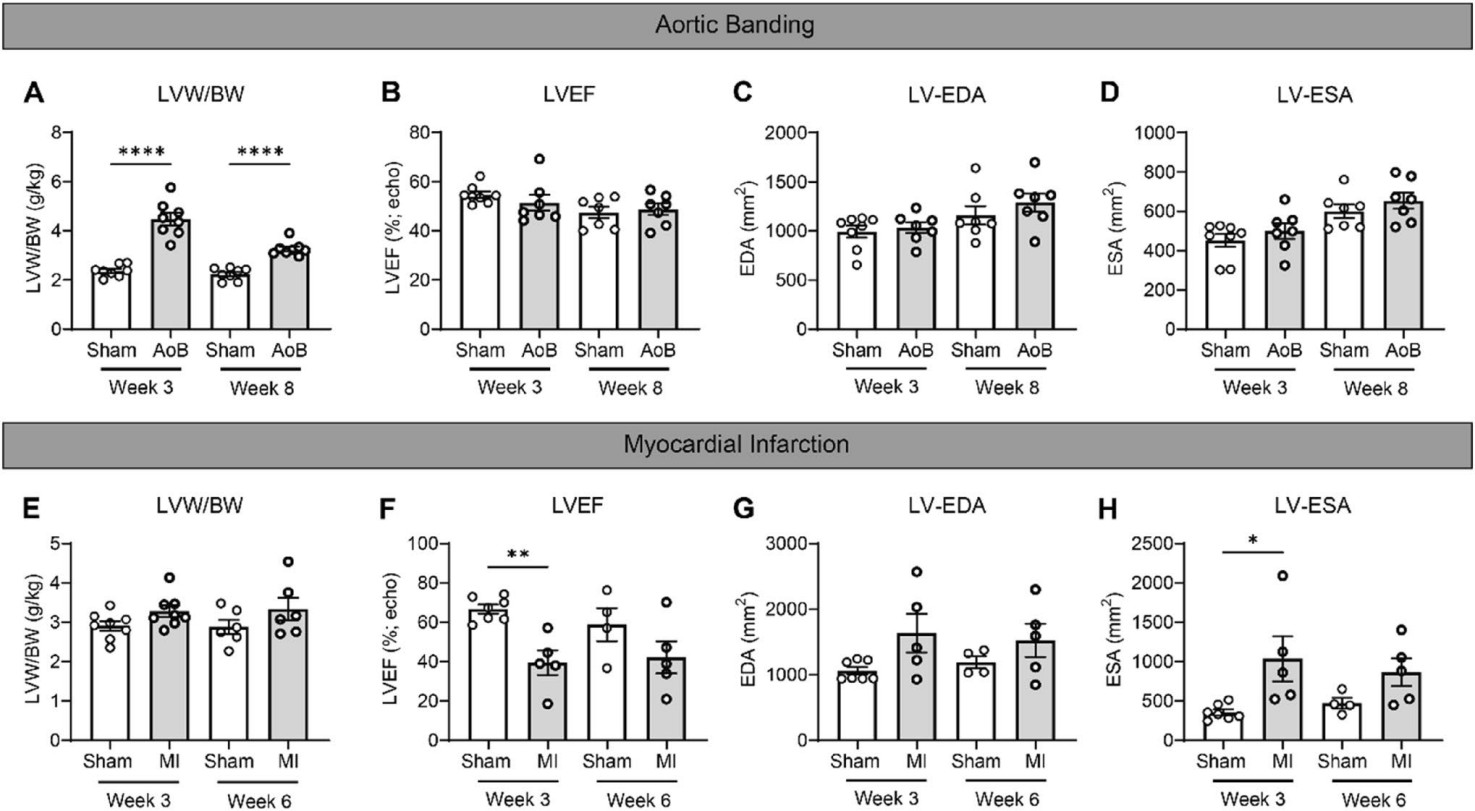
Cardiac characteristics in (**A**–**D**) sham-operated (*n* = 8 at both week 3 and 8) or AoB-treated pigs (*n* = 8 at both week 3 and 8) and (**E**–**H**) sham-operated (*n* = *8* at week 3, *n* = 6 at week 6) or MI pigs (*n* = 8 at week 3, *n* = 6 at week 6). (**A** and **E**) Left ventricular weight/body weight (LVW/BW) ratios. (**B** and **F**) Echo LV ejection fractions (LVEF). (**C** and **G**) LV end-diastolic lumen area (LV-EDA) and (**D** and **H**) normalized LV end-systolic lumen area (LV-ESA). AoB-treated = bold open circles, MI = bold open circles, 4-month-old or sham operated = open circles. Each dot in the scatter plots represents an individual sample. * *P* < 0.05, ** *P* < 0.01, and **** *P* < 0.001 versus sham-operated pigs. Measurements are mean ± SEM

**Fig. 4:**
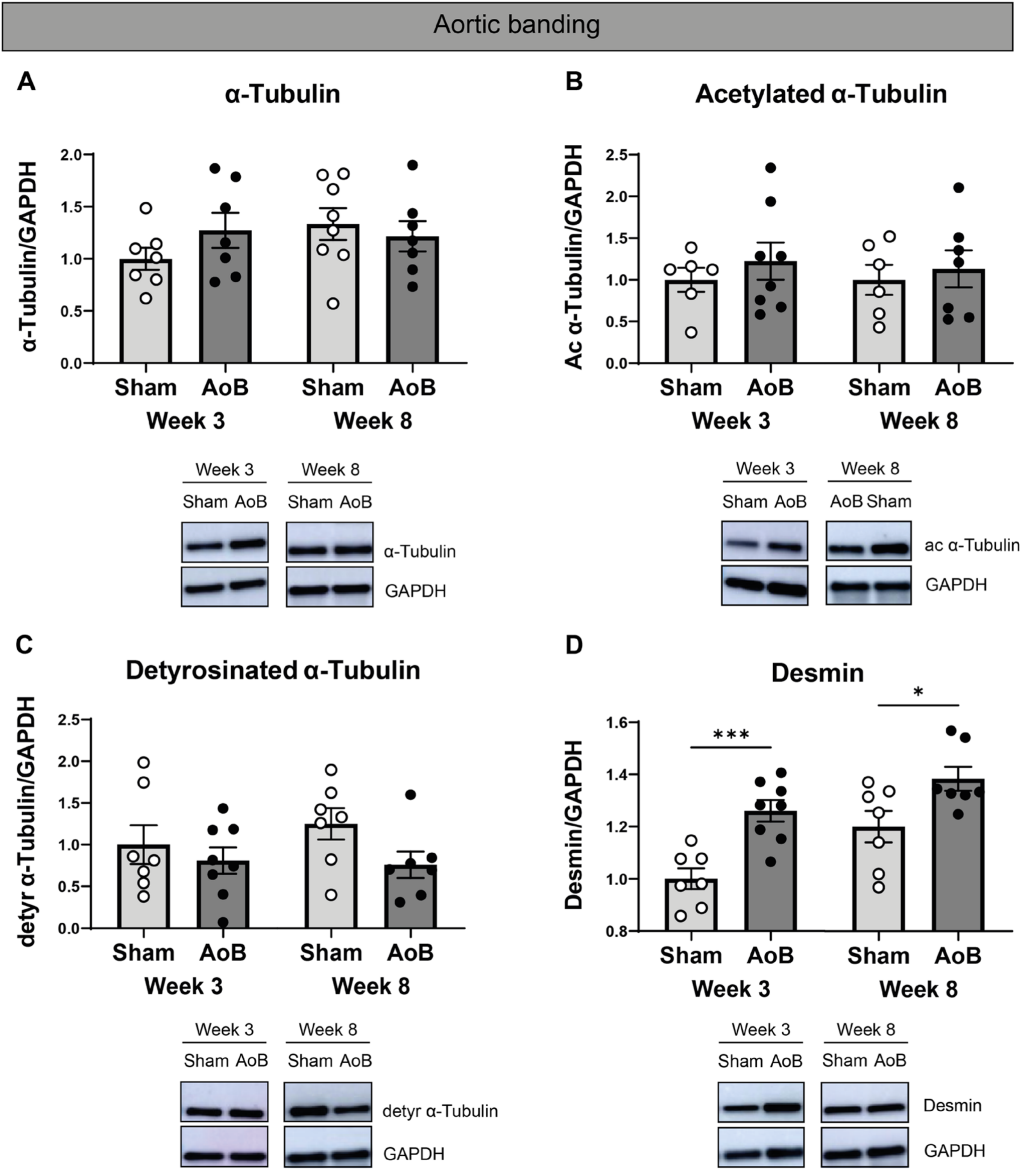
Increased desmin levels in AoB-treated pigs over time. In (**A**–**D**), AoB-treated pigs were compared to sham-operated pigs, with representative western blot images shown accordingly. Quantified levels of (**A**) α-tubulin, (**B**) acetylated α-tubulin, (**C**) detyrosinated α-tubulin, (**D**) and desmin. Sham operated = open circles, AoB-treated = filled circles. Each dot in the scatter plots represents an individual sample. * *P* < 0.05 and *** *P* < 0.001 versus 4 months, sham-operated pigs. Measurements are means ± SEMs

The biobank of previously collected pig samples offered a unique opportunity to assess if concentric or eccentric cardiac remodeling coincides with a change in microtubules and desmin levels and allowed us to define changes at different time points after induction of concentric and eccentric remodeling with AoB and MI, respectively. Figure 3 provides an overview of the baseline characteristics of our pig models. The LVW/BW ratio was increased (*P* < 0.001) in AoB pigs at both 3 and 8 weeks after surgery. In contrast, cardiac function, including LVEF, normalized LV-EDA, and normalized LV-ESA, were comparable in AoB and sham-operated pigs. Levels of total, acetylated, and detyrosinated α-tubulin were not different between AoB and sham-operated pigs at both 3 and 8 weeks after intervention (Figs. 4A–C and Figure S8). Desmin levels, however, were significantly higher at 3 and 8 weeks after AoB compared to sham-operated pigs (Fig. 4D). Interestingly, when normalized to total tubulin levels, detyrosinated α-tubulin was significantly decreased in AoB-treated pigs at 8 weeks after intervention (Figs. S9-A and B).

### Unaltered tubulin signature in pigs with MI-induced eccentric cardiac remodeling

**Fig. 5:**
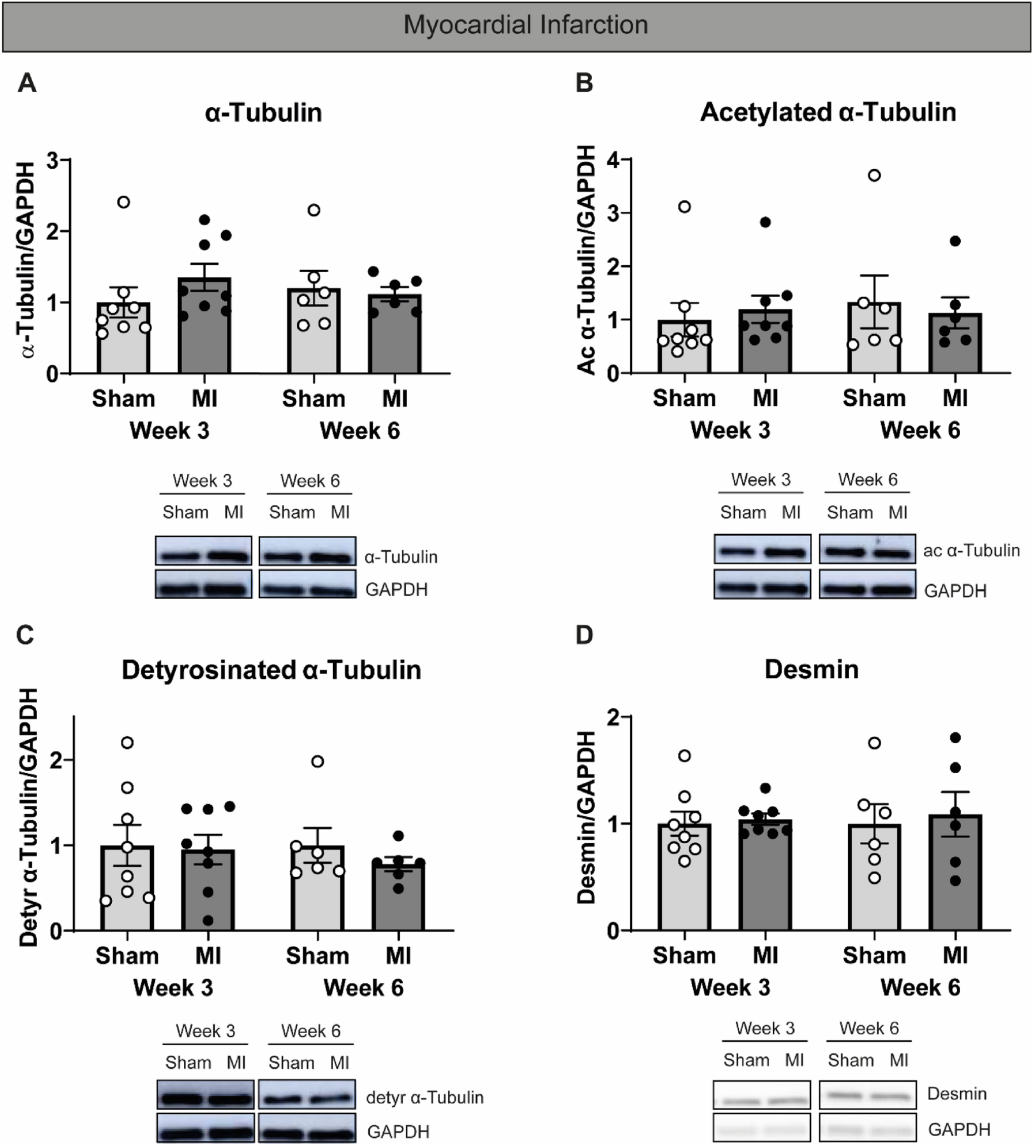
Unaltered levels of microtubules and post-translational modifications in infarcted pigs. In (**A**–**D**), infarcted pigs were compared to sham-operated pigs, with representative western blot images shown accordingly. Quantified levels of (**A**) α-tubulin, (**B**) acetylated α-tubulin, (**C**) detyrosinated α-tubulin, (**D**) and desmin. Sham-operated = open circles, infarcted = filled circles. Each dot in the scatter plots represents an individual sample. Measurements are means ± SEMs

At 3 weeks after the intervention, MI pigs presented with a 27% reduction of LVEF (*P* < 0.01), and a 2.9-fold increase in LV-ESA (*P* < 0.05) (Fig. 3F and H). Six weeks after MI, however, both cardiac remodeling and function in MI pigs were comparable to sham-operated pigs (Fig. 3A–H). Levels of total, acetylated, and detyrosinated α-tubulin and desmin were not different between MI pigs at either 3 or 6 weeks after surgery, compared to sham-operated animals (Figs. 5A–D and S9C and D).

### The age dependency of the tubulin signature in pigs

**Fig. 6:**
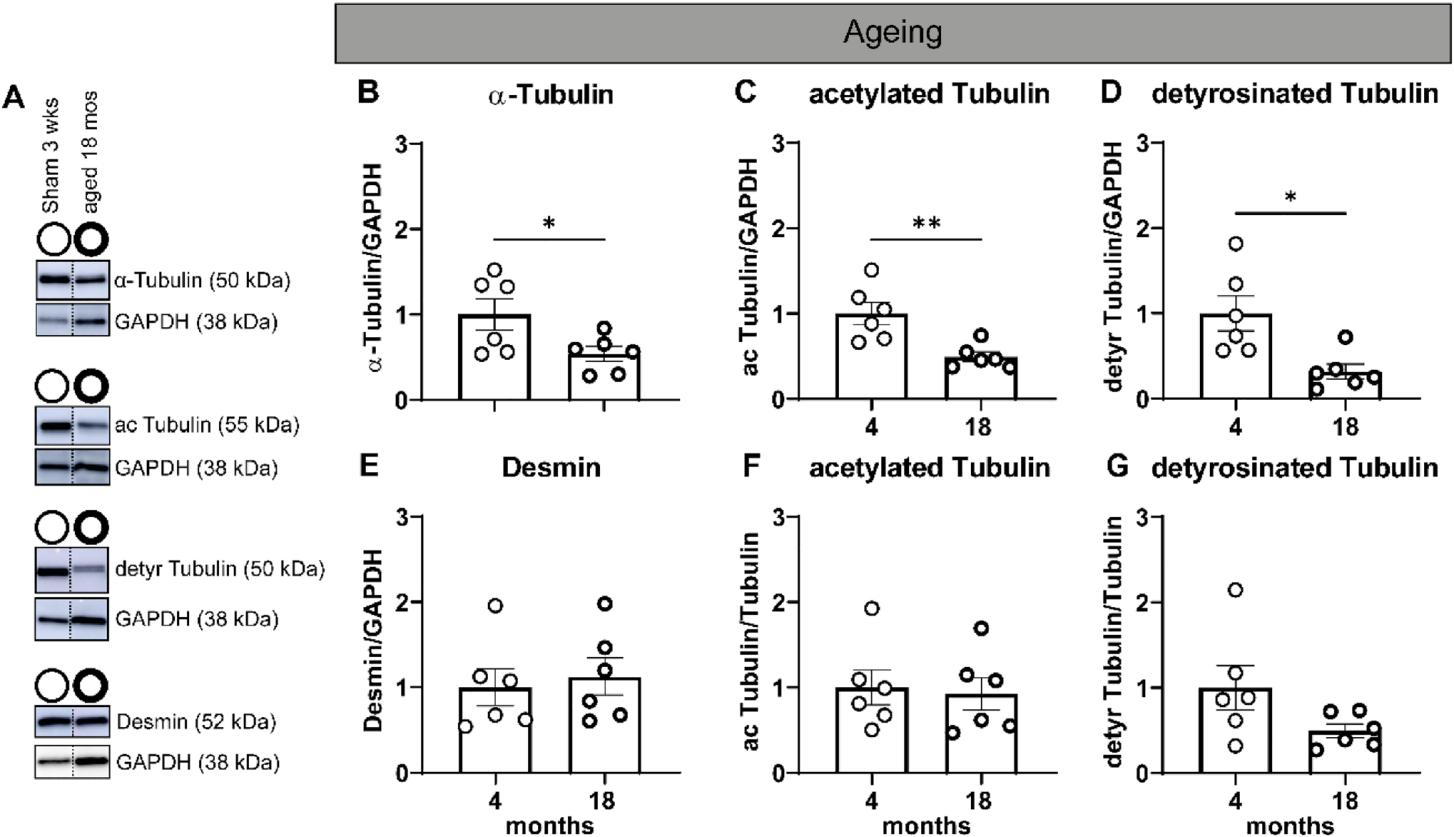
Reduced levels of microtubules and post-translational modifications in adult compared to adolescent pigs; increased desmin levels after AoB intervention. **A** Representative blot images; dashed lines indicate lanes which were run on the same membrane but were noncontiguous. Lanes that are unlabeled constitute protein ladders. Uncropped membranes are presented in Figure S2. In (**B**–**G**), 4-month-old pigs were compared to 18-month-old pigs. Quantified levels of (**B**) α-tubulin, **C** acetylated α-tubulin, **D** detyrosinated α-tubulin, **E** desmin. Due to significant differences in α-tubulin levels, **F** acetylated α-tubulin and **G** detyrosinated α-tubulin were normalized to α-tubulin. 4-month-old, sham operated = open circles and 18-month-old = bold open circles. Each dot in the scatter plots represents an individual sample. * *P* < 0.05 and ** *P* < 0.01 versus 4 months, sham-operated pigs. Measurements are means ± SEMs

We next sought to define the tubulin and desmin signature in 18-month old, i.e., mature, versus 4-month-old pigs, i.e., young adolescent. As was described previously, these mature pigs present with cardiac dysfunction in the absence of LV hypertrophy (Table S2) (van Essen et al. 2018). Compared to young adolescent pigs, we observed reduced levels of α-tubulin (46%; *P* = 0.04), acetylated α-tubulin (51%, *P* = 0.005), and detyrosinated α-tubulin (68%, *P* = 0.01) in mature pigs (Fig. 6A–C). Levels of desmin and acetylated α-tubulin, when normalized to α-tubulin, were not different between both age groups (Fig. 6D and E). In mature pigs, levels of detyrosinated α-tubulin were 50% reduced when normalized to α-tubulin (Fig. 6F).

## Discussion

**Fig. 7:**
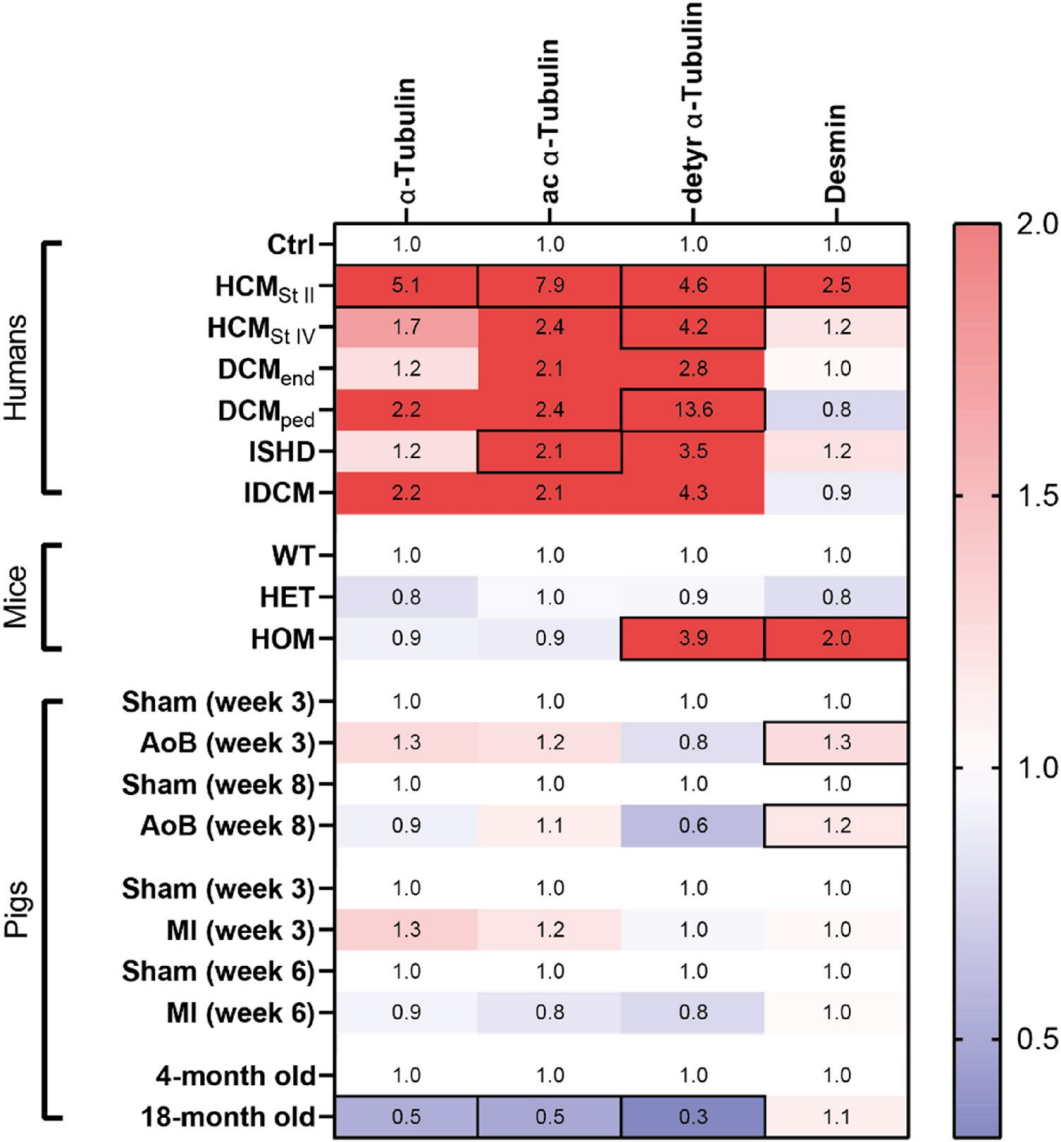
Heat map displaying the mean values of the western blot analyses for the human (Fig. 1), mouse (Fig. 2), and pig samples (Figs. 4–6). Blue indicates lower protein expression and red indicates higher protein expression compared to the respective controls. Mean values above 2 are presented in dark red. Fields containing a statistically significant value are surrounded by boxes

Our analyses of the tubulin signature and desmin expression in a large set of human and pig LV tissues show that the most proliferated and modified tubulin signature and desmin expression is present in patients with obstructive HCM (Fig. 7). No major changes were observed in our large animal models at different time points after AoB and MI surgery. Noteworthy, in the non-failing mature pigs, a decline was observed in the microtubule content compared to young pigs. The latter observation may indicate that an age-related reduction of the microtubular network counteracts disease-mediated cytoskeletal changes in the heart.

### Increased levels of desmin as an early marker of concentric hypertrophy

Since pressure overload was initiated at 3 or 8 weeks before sacrifice, we were able to study the consequences of acute concentric hypertrophy in the absence of any co-founding pathomechanisms such as gene mutations. As expected, AoB-treated pigs were characterized by increased LVW/BW ratios compared to sham-operated pigs. Interestingly, we did not observe any changes in their tubulin signature, which is in line with the fact that the cardiac function, including LVEF, LV-EDA, and LV-ESA was comparable between AoB-treated and sham-operated pigs. Our findings are consistent with previous reports where no microtubule proliferation was observed in guinea pigs (Collins et al. 1996; Wang et al. 1999) with pressure-overload hypertrophy and preserved cardiac function. Other studies modeling pressure-overload hypertrophy (in dogs (Tagawa et al. 1998) and mice (Zhang et al. 2014)) denote cardiac dysfunction alongside microtubule proliferation, though this upregulation may be transient (in cats (Bailey et al. 1997)). In view of this, our MI pigs, which model eccentric hypertrophy, did not present with microtubule proliferation, despite developing cardiac dysfunction. It should be mentioned that although our MI pigs do not present with a statistically significantly increased LVW/BW ratio at 3 weeks, this is likely do to our sample size, as an increased LVW/BW ratio was described previously for these pigs (Kuster et al. 2011). Given the time window, it may be inferred that more time and additional disease triggers are required to recapitulate the complexity of clinical ischemia-induced cardiac remodeling (van der Velden et al. 2022). Thus, the tubulin signature may be reflective of cardiac function and disease stage, with stabilization of microtubules indicating myocardial dysfunction.

In addition to defining the tubulin signature, we assessed the desmin expression in our sample collection. Desmin has been identified as a sarcomeric microtubule anchor (Robison et al. 2016) and is an intermediate filament that cross-links with microtubules in a detyrosination-dependent manner (Gurland and Gundersen 1995; Liao and Gundersen 1998). Compared to sham-operated controls, we detected increased levels of desmin in AoB-treated pigs. Increased levels of desmin were previously suggested to be an indicator of diastolic dysfunction in several HCM mouse models, while we do not observe cardiac dysfunction (Sheng et al. 2016). This upregulation of desmin content accompanying hypertrophy, however, was reported previously (Collins et al. 1996; Hein et al. 2000; Rappaport and Samuel 1988). We, therefore, propose that desmin is an early marker of concentric hypertrophy, instead.

### Tubulin signature in early HCM and end-stage HCM and DCM

Given the tendency of the tubulin signature to align with cardiac functioning, we observed a similar pattern in our clinical samples. The tubulin signature is markedly upregulated in HCM_StII_ patients. In tissue from end-stage HCM patients, detyrosinated α-tubulin levels remain increased, whilst a transient decrease is observed for total and acetylated α- tubulin and desmin. When comparing different end-stage eccentric HF samples, we mostly observed no changes in tubulin signature. Compared to control, acetylated α-tubulin was increased in ISHD patients and detyrosinated α-tubulin in DCM_ped_.

Acetylation (ac) of α-tubulin at Lys40 decreases flexural rigidity of microtubules and thereby protects microtubules from mechanical bending-induced breakage and disassembly (Portran et al. 2017; Xu et al. 2017), but the effect of increased levels of acetylated microtubules on cardiomyocyte function is not yet clear as it was also suggested to increase cardiomyocyte viscoelastic resistance, instead (Coleman et al. 2021). We have also reported increased levels of acetylated α-tubulin in HCM patient samples (Dorsch et al. 2019). In the absence of a functional understanding of tubulin acetylation, future studies are warranted to investigate its significance. Detyrosination, on the other hand, is the removal of tyrosine from the C-terminus of α-tubulin and is linked to increased stiffness and viscoelasticity that, together, reduce contractility (Caporizzo et al. 2018; Chen et al. 2018, 2020; Robison et al. 2016). Previously, we have also reported increased levels of detyrosinated α-tubulin in samples from HCM patients (Schuldt et al. 2021).

Partially contrasting our findings, failing cardiomyocytes of human LV explanted tissues were reported to show microtubule proliferation alongside increased levels of detyrosinated α-tubulin (Chen et al. 2018; Robison et al. 2016). The genotypes of the HCM and DCM patients were not assessed in these studies.

Canonical co-founders such as differences in age ranges, sex, genotype and selection of cardiac regions for biopsies may explain the partially different findings in tubulin signature of HF patients in the various studies. For example, the HF patients with end-stage HCM included in our study were obviously younger than the ones in the studies of Prosser’s group, indicating a possible confounding effect of aging. A description of the tubulin signature in the different regions of the heart is still lacking, but of utmost importance to understand how the selection of a specific region is introducing a bias when comparing different studies.

Furthermore, our data suggest that desmin levels are increased in early concentric hypertrophy, while our end-stage HF patient cohort was characterized by desmin levels comparable to controls. However, compared to non-failing and compensated hypertrophic hearts, upregulation of desmin and some low molecular weight bands that are prone to misfolding, aggregation, and cleavage (Agnetti et al. 2014) were detected in biopsies of not only HCM, but also DCM and ISHD patients with HF (Chen et al. 2018). In addition, explanted failing human myocardium was also reported to show an increase in desmin content (Hein et al. 2000). As is the case with the tubulin signature, several factors, including genotype and disease stage, may contribute to this discrepancy. Since increased desmin levels in HF, independent of etiology, are often accompanied by intracellular desmin aggregates (Agnetti et al. 2014; Singh et al. 2020), further research is warranted to improve our understanding about the extent of these modifications and their functional ramifications in HCM.

### Genotype-dependent tubulin signature in MYBPC3 mice

We did not observe microtubule markers to be more abundant in HET MYBPC3_2373insG_ mice, which is in line with the absence of an HCM phenotype. This unchanged tubulin signature in heterozygous mice differs from our previous study with older HOM *MYBPC3* mice. We observed normal α-tubulin levels and increased levels of detyrosinated α-tubulin and desmin in homozygous, 20 to 28 weeks old *MYBPC3*_2373insG_ mice and in homozygous, 55 to 59 weeks old *MYBPC3*_772G>A_, we observed an increase in α-tubulin and a tendency to increased detyrosination of α-tubulin (Schuldt et al. 2021). Desmin levels were not studied in the latter group.

Taken together, these findings suggest that the tubulin signature and desmin expression may be altered in a time-dependent sequence in response to a genetic trigger. An intermediate stage may be represented by an increase in desmin levels, followed by more detyrosination of α-tubulin and finally microtubule proliferation with a tendency toward increased α-tubulin detyrosination. Further research is needed to understand the age-dependent differences in tubulin signature and desmin expression and their functional ramifications in *MYBPC3*_2373insG_ mice.

### Effect of aging and body growth in parallel

Collectively, our data suggest that the tubulin signature is independent of cardiac remodeling and is reflective of cardiac function instead. In this study, we also used 18-month-old pigs with a high BW. We observed that these pigs have a reduced tubulin signature, which includes decreased levels of total, acetylated, and detyrosinated α-tubulin, yet these pigs present with diastolic dysfunction. According to allometric scaling laws, LV dimensions and function do not scale with BW in modern pigs (van Essen et al. 2018; West and Brown 2005; West et al. 1997). Aging, then, may interact with the disease model in fine tuning the tubulin signature. More research is needed to address the complexity of this relationship. Hereby, it is important to study the effect of AoB and MI in adult pigs (i.e., 2-year-old pigs) as well as including aged adult pigs (i.e., 5-year-old pigs, with minimal or no body growth).

One limitation of our study is the comparison of the 18-month-old pigs to sham-operated, 4-month-old pigs. Due to differences in breeders and times at which the measurements took place, these animals likely have a slightly different genetic background. In addition, the 18-month-old pigs were only females. It would be interesting to determine the tubulin signature in female pigs of the lowest weight category of the van Essen study (van Essen et al. 2018), which is comparable to the weights of the sham-operated pigs.

## Conclusion

Cardiomyopathies underlie complex pathomechanisms and animal models only partially recapitulate the proliferated and modified tubulin signature and changes in desmin levels. Disease etiology and stage, age, sex, and selection of a heart region are likely to introduce bias into the tubulin signature. Our findings emphasize the necessity to establish better model systems to precisely elucidate the effect (compensation or consequence) of the observed HCM and DCM tubulin signatures on contractility, microtubule dynamics, and stability.

### Supplementary Information

Below is the link to the electronic supplementary material.

Supplementary file1 (DOCX 21 kb)

Supplementary file2 (DOCX 17 kb)

## Data Availability

Data sharing is not applicable to this article as no datasets were generated or analysed during the current study.
