# Supplementary material for: The microtubule signature in cardiac disease: etiology, disease stage, and age dependency
Source: J Comp Physiol B. 2023 Aug 29;193(5):581–95. doi: 10.1007/s00360-023-01509-1 (PMC10533615; doi:10.1007/s00360-023-01509-1)
Supplement: Supplementary file 1 — Supplementary file1 (DOCX 21 kb) [file 360_2023_1509_MOESM1_ESM.docx]

# Supplementary Figures


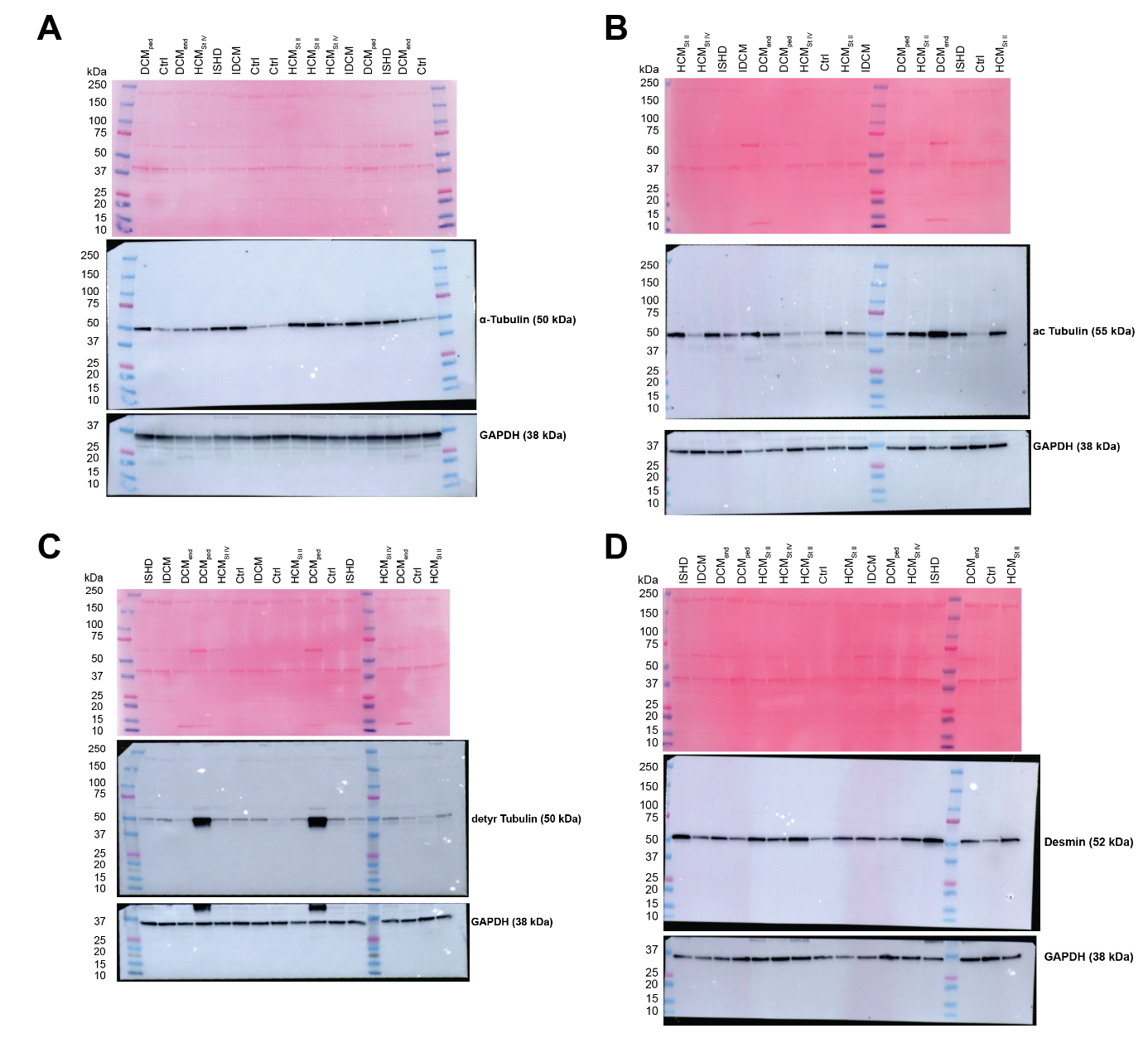


**Figure S1**. Uncropped full-width pictures of Western blotting membranes loaded with human samples. Membranes were usually cut to enable blotting for multiple antibodies. (**a**) Full membrane was stained for α-tubulin and membrane fragment (<45 kDa) was thereafter stained for GAPDH. (**b**) Full membrane was stained for acetylated α-tubulin and membrane fragment (<45 kDa) was thereafter stained for GAPDH. (**c**) Full membrane was stained for detyrosinated tubulin and membrane fragment (<45 kDa) was thereafter stained for GAPDH. (**d**) Full membrane was stained for desmin and membrane fragment (>45 kDa) was stained for GAPDH


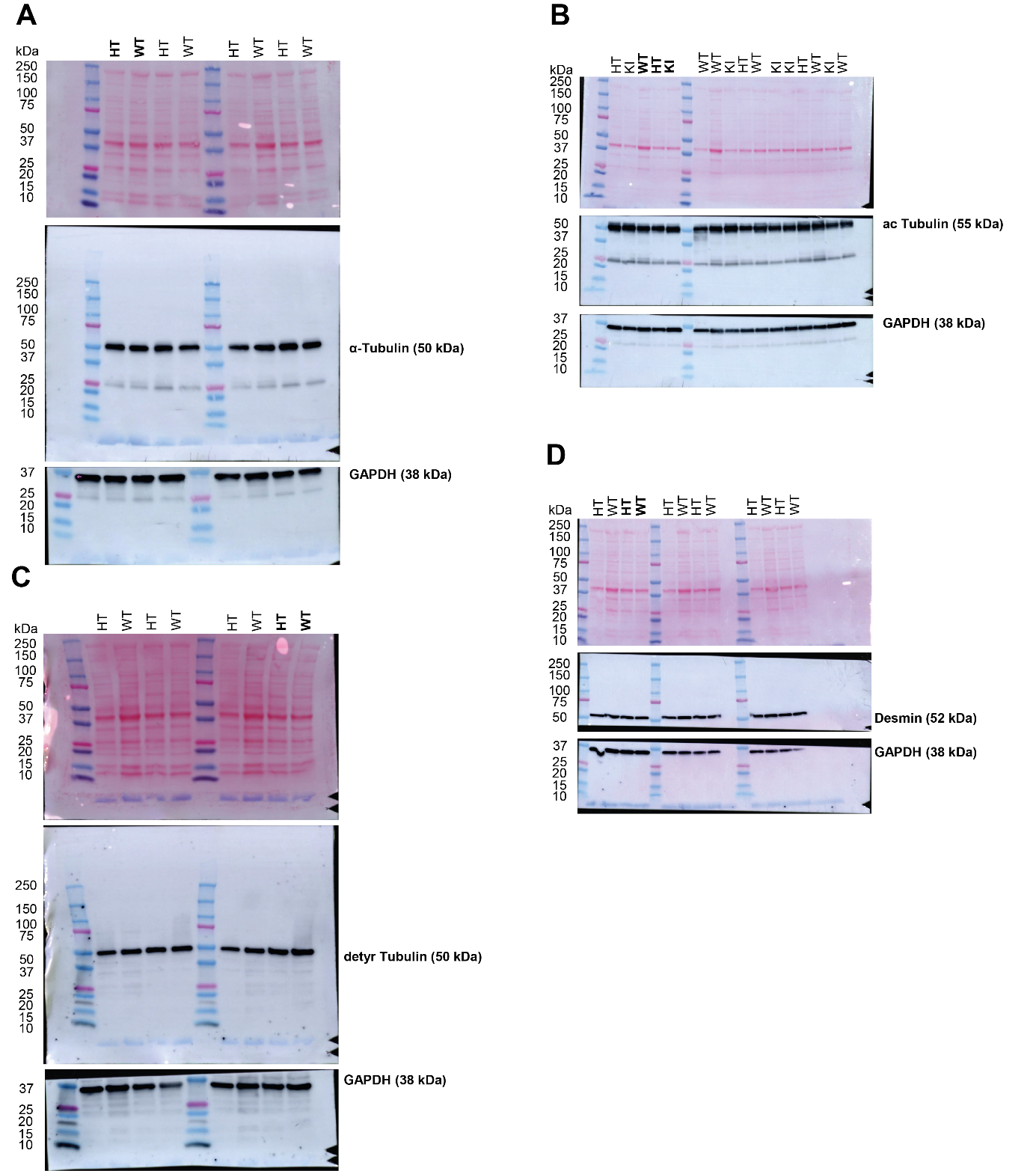


**Figure S2**. Uncropped full-width pictures of Western blotting membranes loaded with mice samples. Membranes were usually cut to enable blotting for multiple antibodies. (**a**) Full membrane was stained for α-tubulin and membrane fragment (<37 kDa) was thereafter stained for GAPDH. (**b**) Cut membrane fragment (10 kDa – 37 kDa) was stained for acetylated α-tubulin and lower membrane fragment was thereafter cut and stained for GAPDH (<37 kDa). (**c**) Full membrane was stained for detyrosinated α-tubulin and membrane fragment (<37 kDa) was thereafter stained for GAPDH. (**d**) Cut membrane fragment (50 kDa – 250 kDa) was stained for desmin and lower membrane fragment (<37 kDa) was stained for GAPDH. Samples in bold typeface indicate the samples discussed in the manuscript


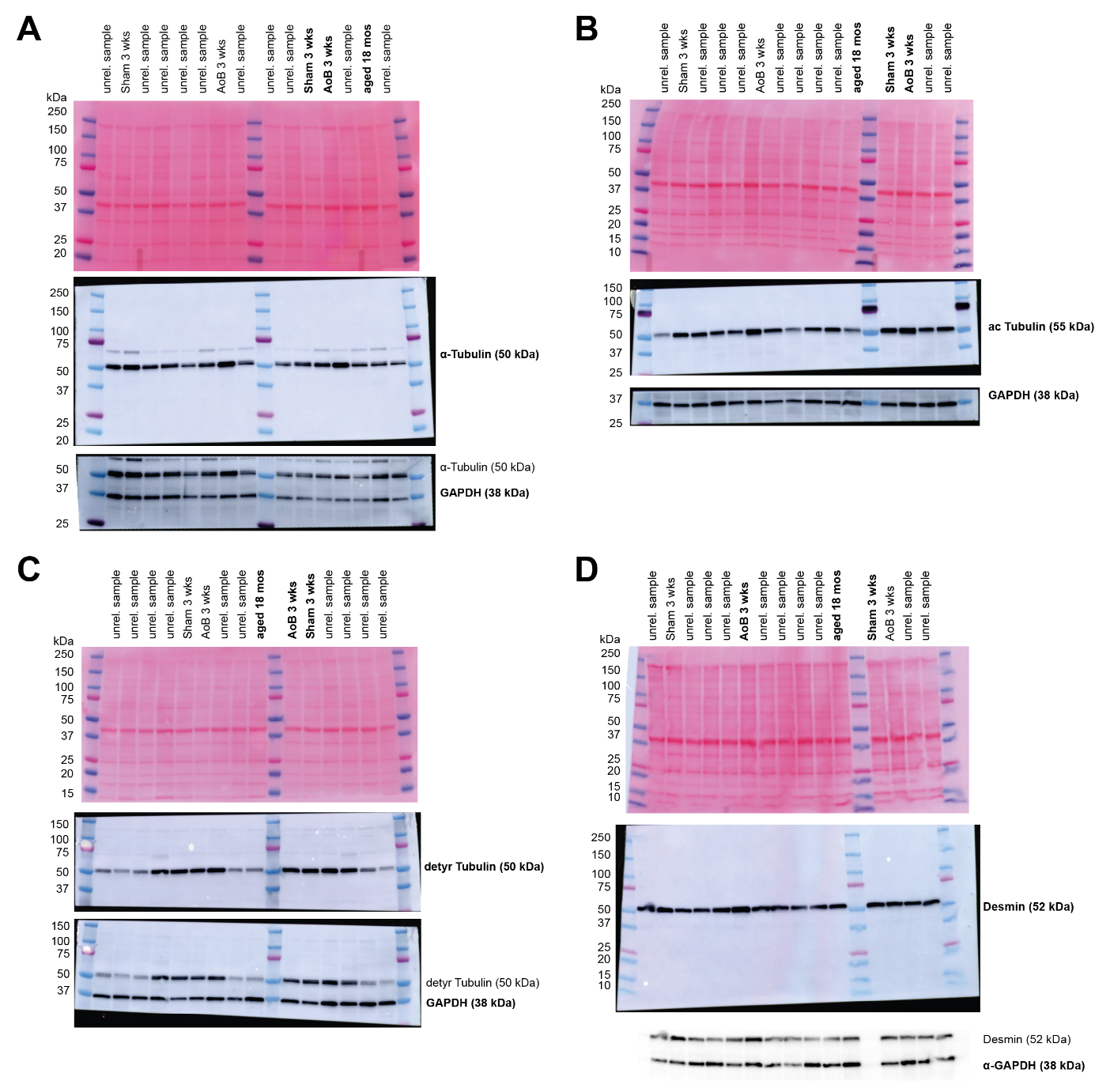


**Figure S3**. Uncropped full-width pictures of Western blotting membranes loaded with pig samples. Membranes were usually cut to enable blotting for multiple antibodies. (**a**) Full membrane was stained for α-tubulin and membrane fragment (>75 kDa) was thereafter stained for GAPDH. (**b**) Cut membrane fragment (25 kDa – 150 kDa) was stained for acetylated α-tubulin . Thereafter membrane was cut at 50 kDa and lower part (<50 kDa) was stained for GAPDH. (**c**) Cut membrane fragment (25 kDa – 150 kDa) was stained for detyrosinated tubulin and thereafter for GAPDH. (**d**) Full membrane was stained for desmin and membrane fragment (25 kDa – 75 kDa) was thereafter stained for GAPDH. Samples in bold typeface indicate the samples discussed in the manuscript


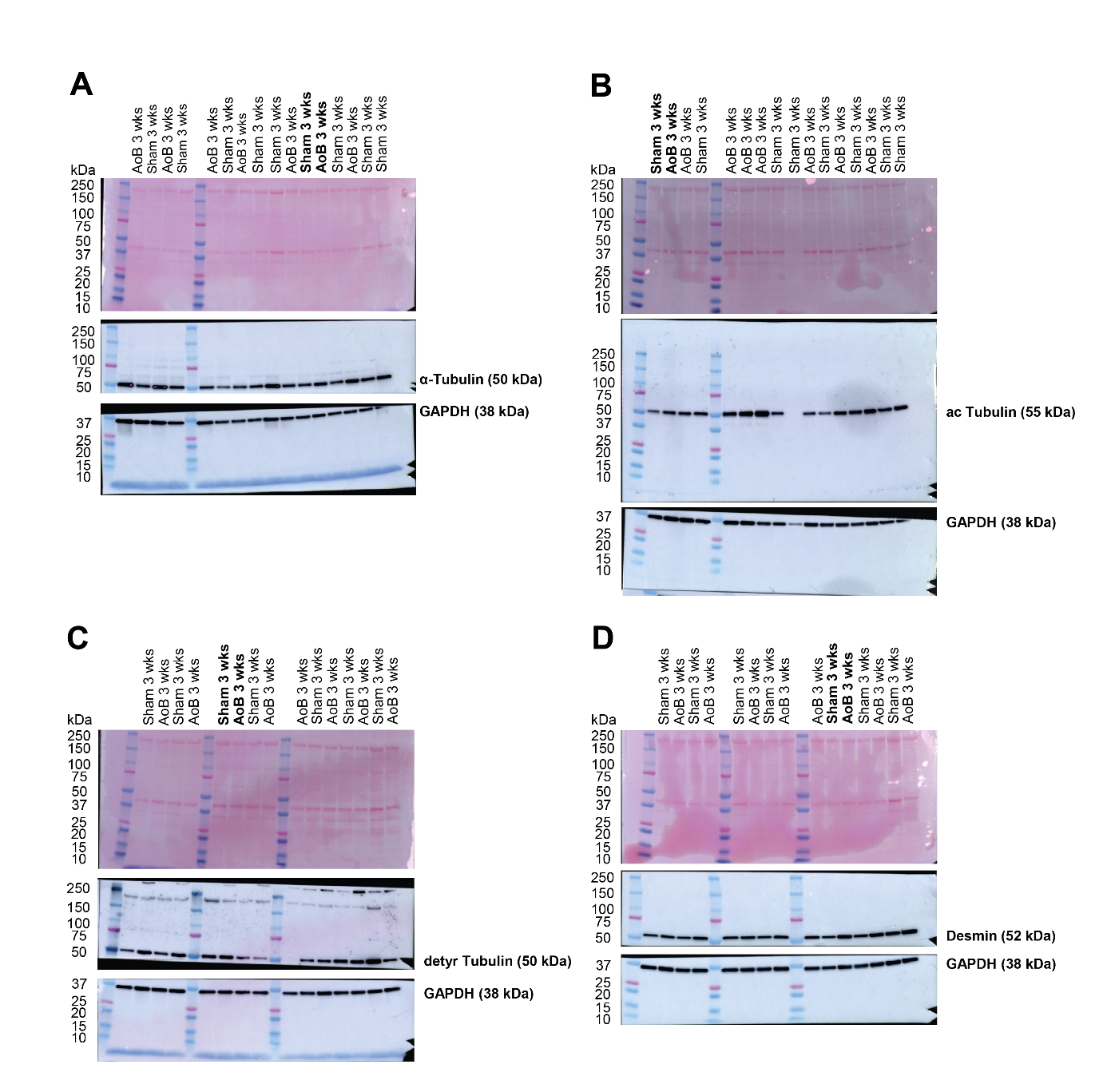


**Figure S4**. Uncropped full-width pictures of Western blotting membranes loaded with AoB-treated pig samples (week 3). Membranes were usually cut to enable blotting for multiple antibodies. (**a**) Cut membrane fragment (50 kDa – 250 kDa) was stained for α-tubulin and lower membrane fragment (<37 kDa) was stained for GAPDH. (**b**) Cut membrane fragment (50 kDa – 250 kDa) was stained for acetylated α-tubulin and lower membrane fragment (<37 kDa) was stained for GAPDH. (**c**) Cut membrane fragment (50 kDa – 250 kDa) was stained for detyrosinated α-tubulin and lower membrane fragment (<37 kDa) was stained for GAPDH. (**d**) Cut membrane fragment (50 kDa – 250 kDa) was stained for desmin and lower membrane fragment (<37 kDa) was stained for GAPDH. Samples in bold typeface indicate the samples discussed in the manuscript


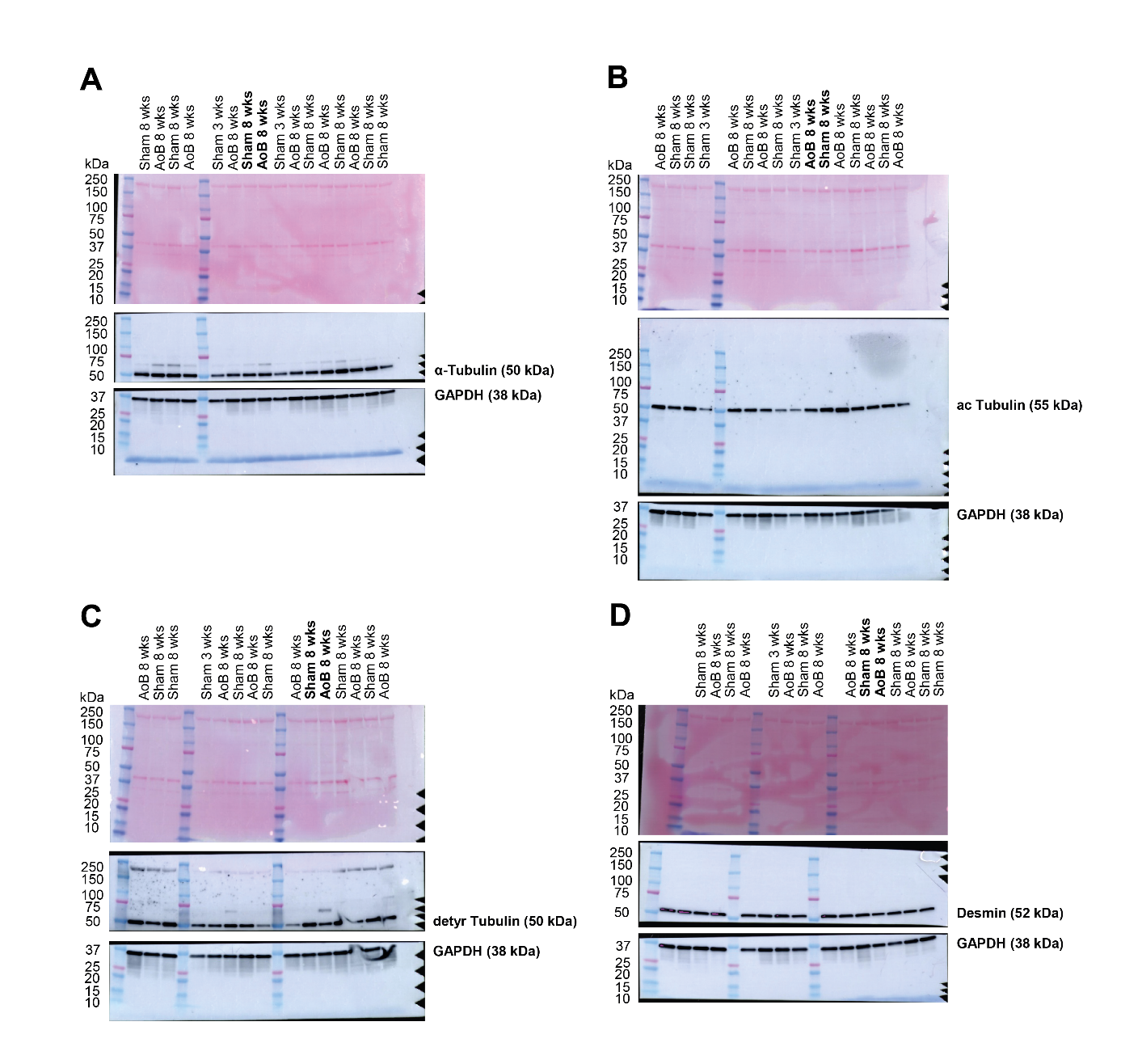


**Figure S5**. Uncropped full-width pictures of Western blotting membranes loaded with AoB-treated pig samples (week 8). Membranes were usually cut to enable blotting for multiple antibodies. (**a**) Cut membrane fragment (50 kDa – 250 kDa) was stained for α-tubulin and lower membrane fragment (<37 kDa) was stained for GAPDH. (**b**) Cut membrane fragment (50 kDa – 250 kDa) was stained for acetylated α-tubulin and lower membrane fragment (<37 kDa) was stained for GAPDH. (**c**) Cut membrane fragment (50 kDa – 250 kDa) was stained for detyrosinated α-tubulin and lower membrane fragment (<37 kDa) was stained for GAPDH. (**d**) Cut membrane fragment (50 kDa – 250 kDa) was stained for desmin and lower membrane fragment (<37 kDa) was stained for GAPDH. Samples in bold typeface indicate the samples discussed in the manuscript


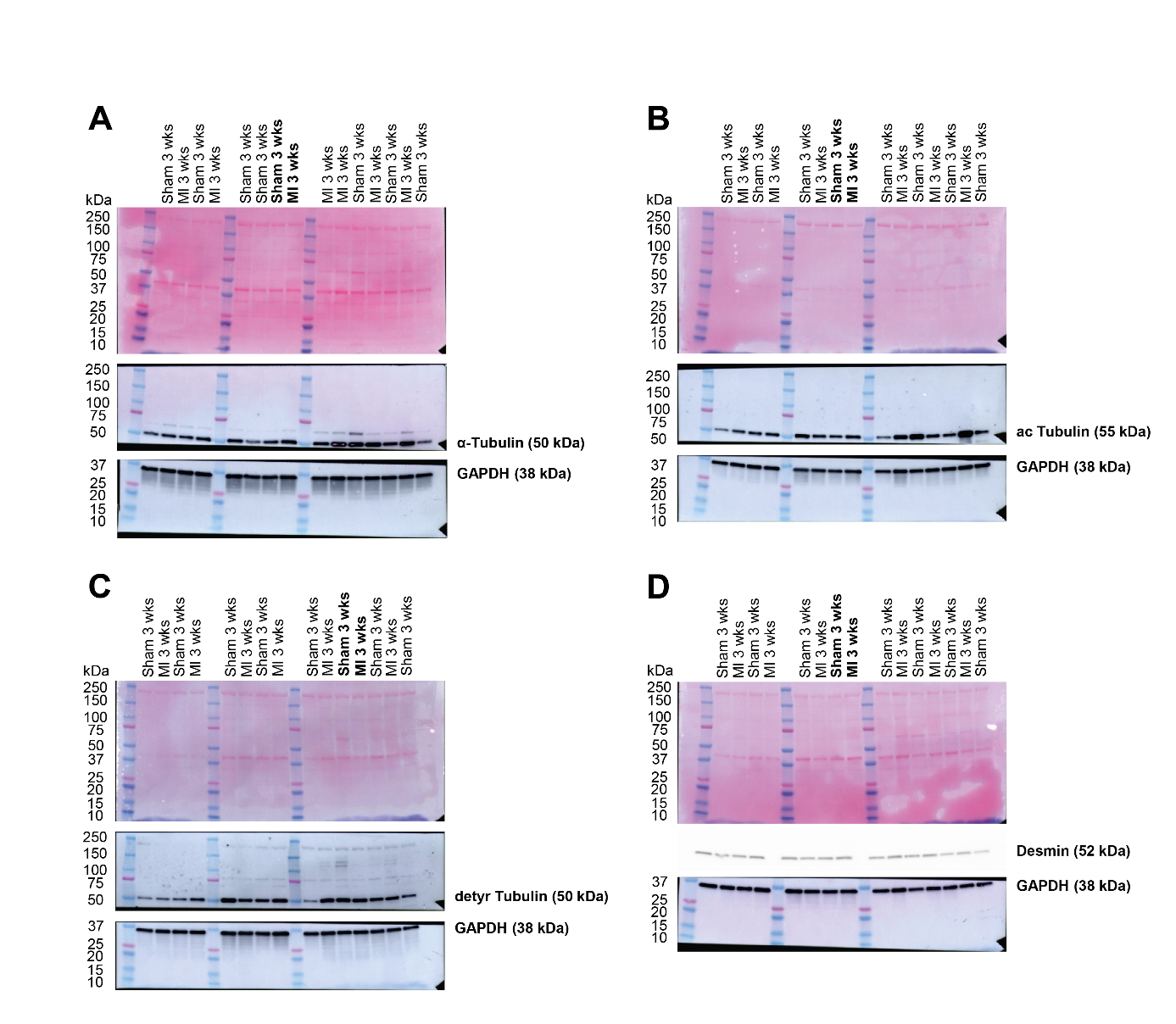


**Figure S6**. Uncropped full-width pictures of Western blotting membranes loaded with infarcted pig samples (week 3). Membranes were usually cut to enable blotting for multiple antibodies. (**a**) Cut membrane fragment (50 kDa – 250 kDa) was stained for α-tubulin and lower membrane fragment (<37 kDa) was stained for GAPDH. (**b**) Cut membrane fragment (50 kDa – 250 kDa) was stained for acetylated α-tubulin and lower membrane fragment (<37 kDa) was stained for GAPDH. (**c**) Cut membrane fragment (50 kDa – 250 kDa) was stained for detyrosinated α-tubulin and lower membrane fragment (<37 kDa) was stained for GAPDH. (**d**) Cut membrane fragment (50 kDa – 250 kDa) was stained for desmin and lower membrane fragment (<37 kDa) was stained for GAPDH. Samples in bold typeface indicate the samples discussed in the manuscript


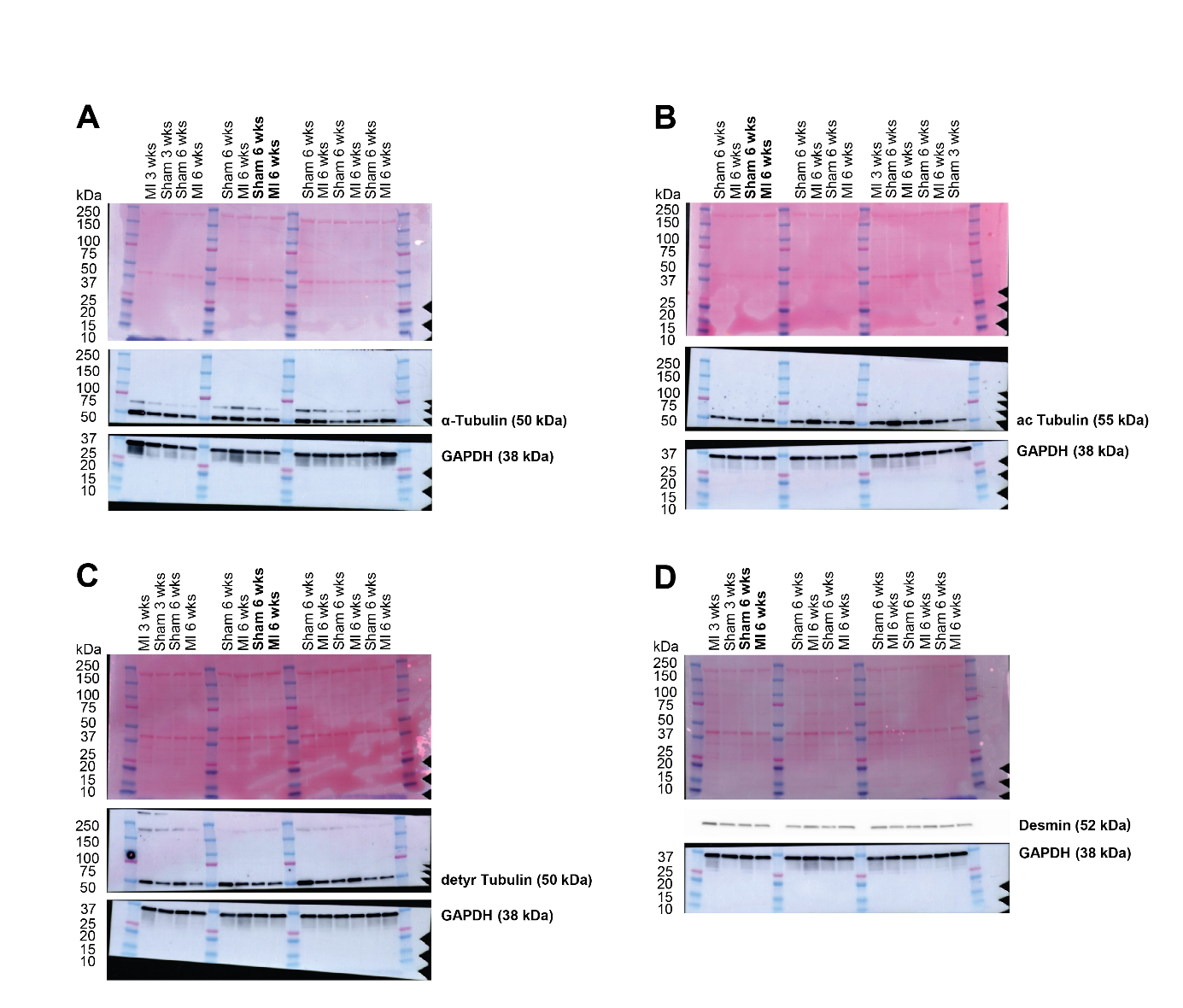


**Figure S7**. Uncropped full-width pictures of Western blotting membranes loaded with infarcted pig samples (week 6). Membranes were usually cut to enable blotting for multiple antibodies. (**a**) Cut membrane fragment (50 kDa – 250 kDa) was stained for α-tubulin and lower membrane fragment (<37 kDa) was stained for GAPDH. (**b**) Cut membrane fragment (50 kDa – 250 kDa) was stained for acetylated α-tubulin and lower membrane fragment (<37 kDa) was stained for GAPDH. (**c**) Cut membrane fragment (50 kDa – 250 kDa) was stained for detyrosinated α-tubulin and lower membrane fragment (<37 kDa) was stained for GAPDH. (**d**) Cut membrane fragment (50 kDa – 250 kDa) was stained for desmin and lower membrane fragment (<37 kDa) was stained for GAPDH. Samples in bold typeface indicate the samples discussed in the manuscript


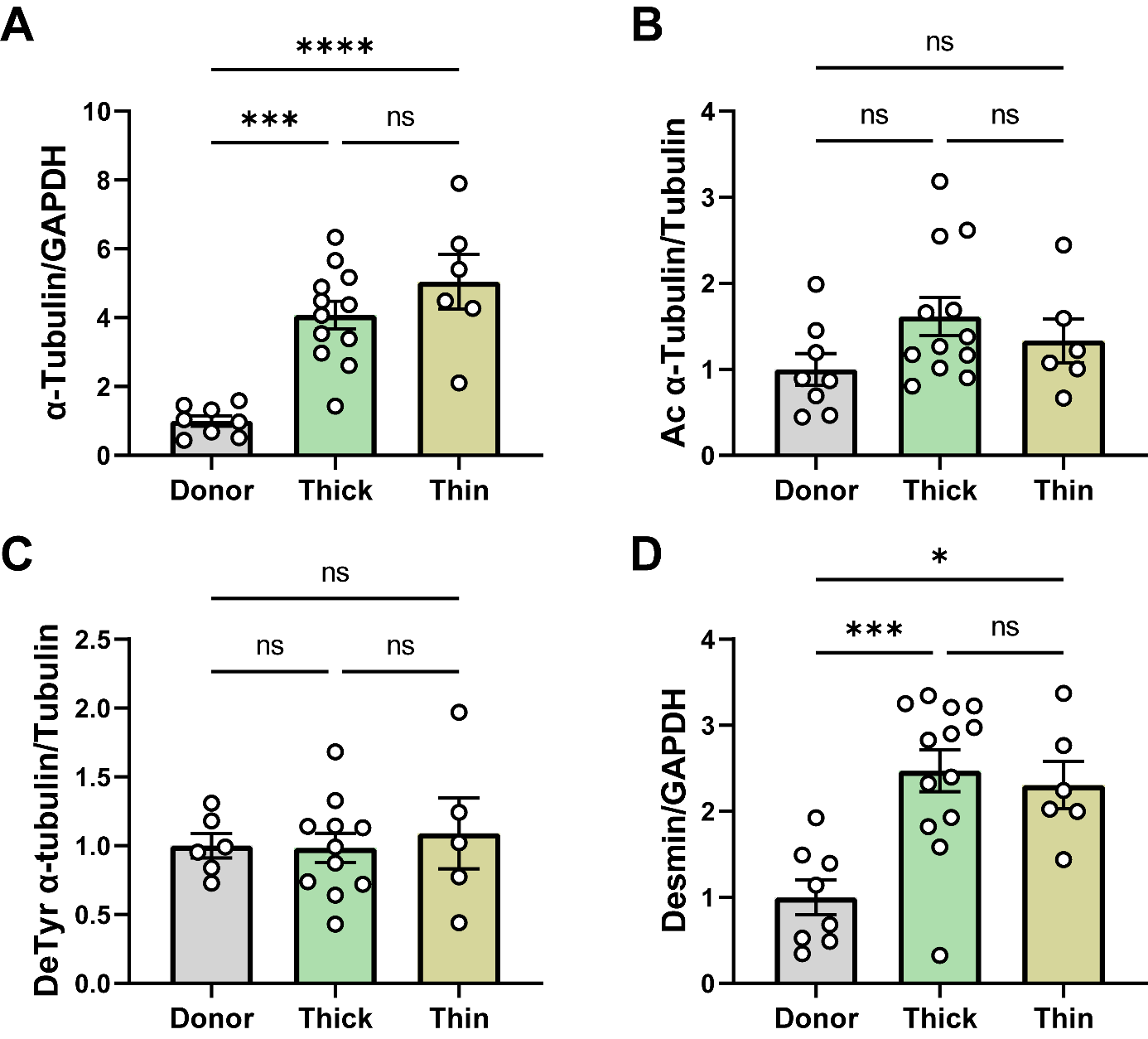


**Figure S7**. The tubulin code in thick (MYH7/MYL2) and thin (TNNI3/TNNT2) mutation-carriers at NYHA Stage II. In (A-D), early (HCM_St II_) HCM samples with either thick or thin filament mutations were compared to non-failing donors. Quantified levels of (A) α-tubulin, (B) acetylated α-tubulin normalized α-tubulin, (C) detyrosinated α-tubulin normalized α-tubulin, and (D) desmin. Each dot in the scatter plots represents an individual sample. Measurements are means ± SEMs


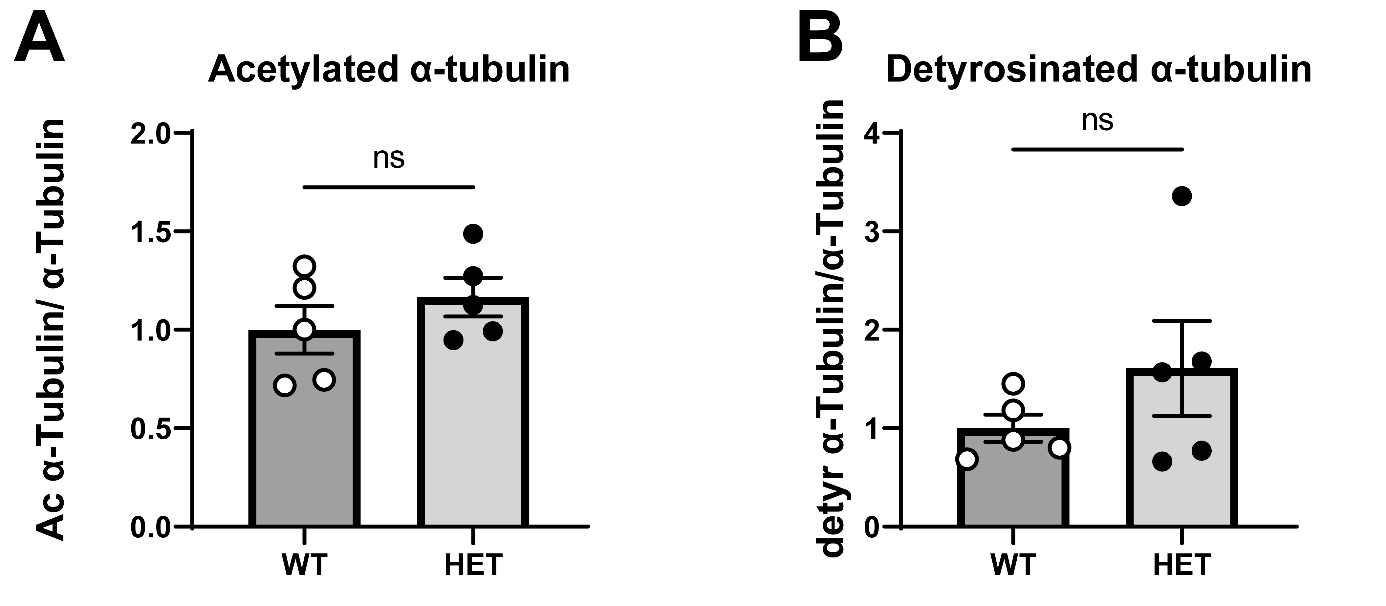
**Figure S8**. Levels of microtubules and posttranslational modifications in heterozygous (HET) MYBPC3 mice remain unaltered. Quantified levels of (A) acetylated α-tubulin and (B) detyrosinated α-tubulin normalized to total α-tubulin. Wild-type (WT) = open circles and HET = filled circles. Each dot in the scatter plots represents an individual sample. Measurements are mean ± SEM


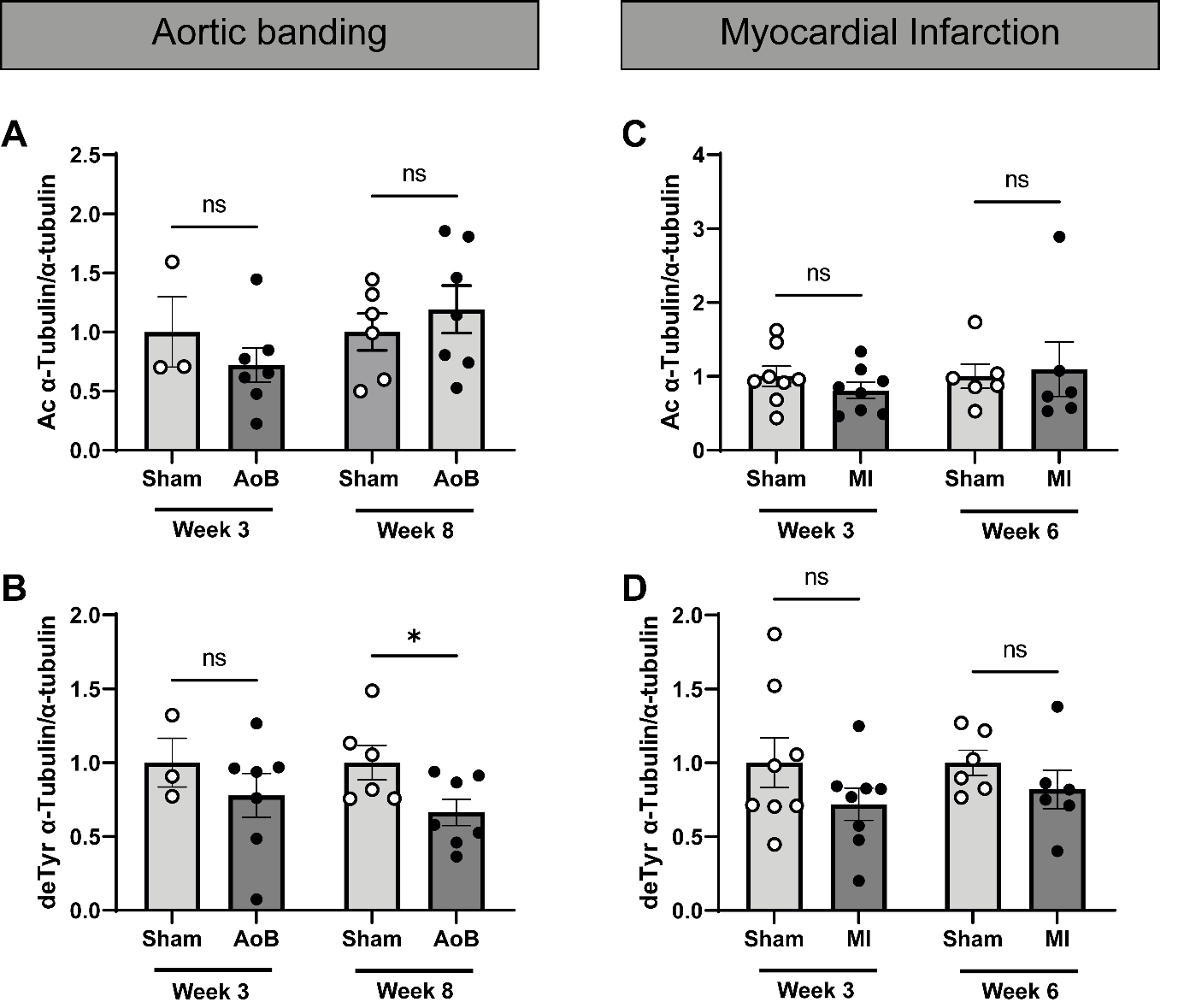


**Figure S9**. Levels of microtubules and posttranslational modifications in aortic banding (AoB) and infarcted (MI) pigs. Quantified levels of (**A** and **C**) acetylated α-tubulin and (**B** and **D**) detyrosinated α-tubulin normalized to total α-tubulin. Sham-operated = open circles, AoB-treated or infarcted = filled circles. Each dot in the scatter plots represents an individual sample. Measurements are means ± SEMs

**Figure S10**. Normalized GAPDH counts in hypertrophic cardiomyopathy (HCM) patients. Measurements are means ± SEMs (Schuldt et al., 2021)
