# Supplementary material for: The microtubule signature in cardiac disease: etiology, disease stage, and age dependency
Source: J Comp Physiol B. 2023 Aug 29;193(5):581–95. doi: 10.1007/s00360-023-01509-1 (PMC10533615; doi:10.1007/s00360-023-01509-1)
Supplement: Supplementary file 2 — Supplementary file2 (DOCX 17 kb) [file 360_2023_1509_MOESM2_ESM.docx]

**Supplementary Tables**

| **Number** | **Group** | **Age (weeks)** | **Sex** | **LVW/BW** | **EDA** | **ESA** | **LVEF (%)** |
| --- | --- | --- | --- | --- | --- | --- | --- |
| 5 | WT | 26,6 ± 0,2 | 3 F, 2 M | 3,8 ± 0,4 | 3,6 ± 0,1 | 2,2 ± 0,1 | 70 ± 2 |
| 5 | HET | 25,8 ± 0,7 | 2 F, 3 M | 3,8 ± 0,2 | 3,5 ± 0,1 | 2,1 ± 0,2 | 73 ± 4 |
| 5 | HOM | 24,6 ± 0,7 | 2 F, 3 M | 7,7 ± 0,5 | 4,9 ± 0,6 | 4,6 ± 0,5 | 14 ± 1 |

**Table S1** Characteristics of the MYBPC3-targeted mouse model.

Age: age at sacrifice; F: female; M: male; EDA: end-diastolic lumen area; ESA: end-systolic lumen area; HET: heterozygous; HOM; homozygous; LVEF: left ventricular ejection fraction; LVW/BW: left ventricular weight/bodyweight; WT: wild-type. Measurements are means ± SEMs

| **Number** | **Group** | **Weeks** | **Sex** | **LVW/BW** | **LV-EDA** | **LV-ESA** | **LVEF (%)** |
| --- | --- | --- | --- | --- | --- | --- | --- |
| 8 | Sham | 3 | 4 F, 4 M | 2,4 ± 0,1 | 993,1 ± 61,1 | 451,5 ± 33 | 55 ± 1 |
| 8 | AoB | 3 | 4 F, 4 M | 4,5 ± 0,3 | 1033,4 ± 54,8 | 499,9 ± 39,8 | 51 ± 3 |
| 8 | Sham | 8 | 4 F, 4 M | 2,2 ± 0,1 | 1159,3 ± 95,1 | 600,5 ± 34 | 47 ± 2 |
| 8 | AoB | 8 | 4 F, 4 M | 3,3 ± 0,1 | 1289,9 ± 94 | 653,9 ± 40,8 | 49 ± 2 |
| 8 | Sham | 3 | 4 F, 4 M | 2,9 ± 0,1 | 1061,2 ± 56 | 355,9 ± 36,5 | 67 ± 2 |
| 8 | MI | 3 | 4 F, 4 M | 3,3 ± 0,1 | 1633,3 ± 295 | 1036,1 ± 284,7 | 39 ± 6 |
| 6 | Sham | 8 | 5 F, 1 M | 2,9 ± 0,2 | 1191,9 ± 92,4 | 470,8 ± 67,1 | 59 ± 8 |
| 6 | MI | 8 | 3 F, 3 M | 3,3 ± 0,3 | 1523,4 ± 253,2 | 862,7 ± 175,2 | 42 ± 8 |
| 6 | Mature |  | 6 F | 2 ± 0,1 | 2821,5 ± 198,9 | 1583,6 ± 154,4 | 44 ± 4 |

**Table S2** Characteristics of pig models.

AoB: aortic banding; MI: myocardial infarction; F: female; M: male; LVEF: left ventricular ejection fraction, calculated as (EDA−ESA)/EDA*100%; LV-EDA: left ventricular end-diastolic lumen area, normalized according to EDV = 41 x BW^0.37; LV-ESA: left ventricular end-systolic lumen area, normalized according to ESV = 41 x BW^0.37; LVW/BW: left ventricular weight/bodyweight, normalized according to LV weight = 0.6 x BW^0.78; Sham: sham-operated; Weeks; number of weeks after intervention. Measurements are means ± SEM
